# The parent–child relationship and child shame and guilt: A meta‐analytic systematic review

**DOI:** 10.1111/cdev.14212

**Published:** 2025-01-16

**Authors:** Rahel L. van Eickels, Magdalena Siegel, Alice J. Juhasz, Martina Zemp

**Affiliations:** ^1^ Department of Clinical and Health Psychology University of Vienna Vienna Austria

**Keywords:** parenting, research synthesis, self‐conscious emotions

## Abstract

Empirical findings on the associations of positive and dysfunctional parent–child relationship (PPCR/DPCR) characteristics with child shame, adaptive guilt, and maladaptive guilt were synthesized in six meta‐analyses. The 65 included samples yielded 633 effect sizes (*N*_total_ = 19,144; *M*_age_ = 15.24 years; 59.0% female; 67.7% U.S. samples, *n* = 12,036 with 65% White, 12.3% Hispanic and Latinx, 10.8% Black, 6.3% mixed race, 5.6% Asian American, 0.3% Native American participants). Small positive correlations were found between DPCR and shame (*r* = .17), DPCR and maladaptive guilt (*r* = .15), and PPCR and adaptive guilt (*r* = .14). A small negative correlation was found between PPCR and shame (*r* = −.12). Sample and study moderators and sources of bias are investigated and discussed.

**Abbreviations**
CHE: correlated and hierarchical effects
DPCR: dysfunctional parent–child relationship
MMAT: Mixed Methods Appraisal Tool
PCR: parent–child relationship
PPCR: positive parent–child relationship
RCT: randomized controlled trial
RVE: robust variance estimation

The capacity to experience self‐conscious emotions, such as shame and guilt, emerges in the early years of life and continues to evolve over the course of childhood with cognitive maturation (Lagattuta & Thompson, 2007). The parent–child relationship, as the child's primary relational and socialization context, may be crucial for the development of self‐conscious emotions and the extent to which these are experienced (Donenberg & Weisz, 1998; Mills, 2005). To address the current lack of a formal synthesis on this subject, the aim of this meta‐analytic systematic review is to summarize the existing research on the associations between parent‐child relationship characteristics and child (nonpathological) shame and guilt.

## Shame and guilt: Adaptive and maladaptive manifestations

Shame and guilt are foremost internal, discrete, self‐conscious emotional reactions elicited by external stimuli to protect the social self (Dickerson et al., 2004; Tracy & Robins, 2004). One of the most established theories describes and distinguishes shame and guilt based on their attributions of felt defectiveness (Lewis, 1971). According to this framework, shame is felt when the *whole self* is judged to be flawed, whereas guilt is felt if an *action* is evaluated as faulty. As a consequence, shame is accompanied by feelings of worthlessness and exposure, whereas guilt is usually associated with regret (Tangney et al., 1996). While both emotions are considered important for interpersonal processes (Dempsey, 2017), within this definition, guilt is deemed a more adaptive response to social transgressions compared to shame, as it often leads to reparative actions and behavioral changes (Tignor & Colvin, 2017). However, if guilt extends beyond what is situationally appropriate and is overly strong, it can also become maladaptive (Carnì et al., 2013; Malti, 2016). Because these conceptualizations of guilt differ quite substantially, we distinguish adaptive and maladaptive guilt entirely for the present review. Shame, on the other hand, is perceived as more global and hurtful than guilt and thus less easily or not reparable (Tangney et al., 1996; Tracy & Robins, 2004). Consequently, shame may cause the actor to withdraw from the shameful situation, suppress the emotions, externalize the blame, or attack oneself or others for the purpose of protecting the self (Elison et al., 2006; Pivetti et al., 2016). While occasional experience of shame might be normative (Dempsey, 2017), on a trait‐like level, shame is considered a maladaptive emotion (Tignor & Colvin, 2017).

While shame and guilt emerge as immediate emotional states, both may become dispositional (Tignor & Colvin, 2017). Some individuals may frequently experience shame and guilt in ambivalent situations, reflecting these individuals' shame‐ or guilt‐proneness as relatively stable traits (Tangney, 1996). Shame and guilt may further become “internalized,” where they are conceptualized as overarching feelings devoid of a specific context (Kim et al., 2011; for a critical notion see Leeming, and Boyle, 2004). The different underlying constructs inform their respective operationalization and cannot be used interchangeably without careful evaluation of potential differential effects. Against this backdrop, the current meta‐analysis distinguishes between studies that assessed guilt and shame at the trait versus state level and between different conceptualizations of these constructs (shame‐/guilt‐proneness, internalized shame/guilt).

In contrast to adaptive guilt, both shame‐proneness and maladaptive guilt are considered as risk factors for psychopathology (Levinson et al., 2016; Muris & Meesters, 2014), but excessive shame and guilt can also represent psychopathological symptoms in themselves (Malti, 2016). The present review focuses solely on nonpathological shame and guilt to distinguish them from pathological aspects.

## Development of self‐conscious emotions

Although the social‐cognitive development of children and adolescents encompasses decades of research, empirical research on the developmental course and changes in shame and guilt over time remains considerably scarce (Orth et al., 2010). Except for a few prospective studies (e.g., Mills, 2003; Mills et al., 2010; Parisette‐Sparks et al., 2017), the longitudinal developmental trajectories of these emotions across childhood and adolescence have not yet been thoroughly investigated. Self‐conscious emotions can only emerge once a child has developed self‐awareness and is able to recognize external standards, which occurs typically within the first 24 months of development (Tracy & Robins, 2004). Shame and guilt become more frequent and complex as children and adolescents start to internalize norms and self‐images and mature in their social‐cognitive development (Lagattuta & Thompson, 2007). Thus, the experience of shame and guilt are part of the normative emotional repertoire (Tracy & Robins, 2004), but with inter‐individual variability in their experienced frequency and intensity (Tangney et al., 2009). Some of this variability might be explained by external factors, such as experiences within the parent‐child relationship (Mills, 2005; Szentágotai‐Tătar et al., 2015).

## The parent‐child relationship and child shame and guilt

A warm, supportive, and caring parent‐child relationship is fundamental for children's healthy development (Cooke et al., 2022), while negative, harsh, and punitive parenting is a risk factor for child mental health problems (Pinquart, 2017). Furthermore, the parent‐child relationship is also a correlate of children's emotional development and experiences (Cooke et al., 2019; Grusec, 2011), including shame and guilt (Parisette‐Sparks et al., 2017; Thompson, 2014). While an overarching theory is lacking, psychodynamic, cognitive‐behavioral, and family system theories all include social relationships as a primary developmental component of shame and guilt, even if they place different emphasis on the parental role (for more detailed literature reviews, see Barrett, 1998; Mills, 2005; Reimer, 1996). Derived primarily from attachment and cognitive‐behavioral theory, but still similar across theories, it is posited that children internalize knowledge about themselves and their social environment from the relationship with their primary caregivers over time, which in turn informs their self‐image in relation to others (Malti, 2016; Mills, 2005).

Considering the differing phenomenology of shame and guilt, it is reasonable to assume that dysfunctional experiences in the parent‐child relationship might be particularly associated with shame‐proneness and maladaptive guilt (Mills, 2005; Thompson et al., 2003). In her review of the empirical research, Mills (2005) emphasized the role of dysfunctional relational experiences with parents or primary caregivers in children's development of increased shame through insecure attachment, emotion socialization, and shaming experiences. Severe forms of negative parenting, such as child neglect, maltreatment, and rejection, are also associated with shame‐proneness and maladaptive guilt (e.g., Bennett et al., 2010; Donohue et al., 2020; Mills, 2005). Adaptive guilt, conversely, is associated rather with positive experiences in the parent‐child relationship (e.g., Gülseven et al., 2022; Meesters et al., 2017). However, as some studies found no such effects or even found the opposite (e.g., Kochanska et al., 1996; Rote et al., 2021), it is necessary to clarify the magnitude, direction, and significance of effects reported within the empirical literature.

## Moderators of the link between the parent‐child relationship and child shame and guilt

In addition to examining overall effects, the moderating influence of various study and sample characteristics has to be considered. For instance, child age is a potential moderator, as shame and guilt develop throughout childhood and adolescence (Reimer, 1996) and the parent‐child relationship changes over time with the fluctuating processes of child development (Boele et al., 2020). Child gender is also assumed to modulate effects, since female children and adolescents show higher state and trait shame compared to their male peers (Bybee, 1998; Reimer, 1996), and the causes of guilt and specific reactions likewise differ between genders (Bybee, 1998). In adult age, female gender is further associated with the greater experience of shame and guilt compared to male gender (Else‐Quest et al., 2012). Gender effects have also been found in parenting research (Endendijk et al., 2016; Morawska, 2020). Moreover, given that approaches to parenting have changed both qualitatively and quantitatively over the last decades (Trifan et al., 2014), the year of publication of a study may be indicative of specific cohort effects, while the observation of a decline in effect sizes over time might also indicate publication bias (Pietschnig et al., 2019). While shame and guilt are considered universal emotions (Tracy & Matsumoto, 2008), the expression, understanding, and appraisal of them across cultures may differ from the Western‐European theoretical standpoint this work was grounded on (Lewis, 1971). In addition, culture might further affect parenting behaviors and values (for a more detailed account on Asian and Asian American parenting, see, e.g., Ng & Wang, 2019). Thus, the geographical region of data collection and the ethnicity of participants should be investigated to account for cultural variability (Wong & Tsai, 2007).

Several methodological aspects that might affect the associations of our core constructs also need to be considered. General shortcomings in the quality of studies might bias the findings. Self‐report measures of shame‐ or guilt‐proneness are available for the ages of 6–8 years onwards (Tangney et al., 1990), but behavioral observation is the method of choice for assessment in younger children (Barrett, 1998). Lear et al. (2022) recently pointed out validity problems in measures of shame as well as theoretical differences between different measures. Furthermore, self‐reports of children and their parents on parental behavior or child emotions do not always align (Michels et al., 2013; Tein et al., 1994), indicating potential confounding effects of measurement instruments or raters for both emotions and parenting. Considering the distinct implications of state versus trait shame and guilt (e.g., proneness), it is crucial to evaluate their operationalization as potential moderators (Dempsey, 2017). In addition, since dispositional shame and guilt are not uniformly operationalized throughout the literature, we investigate possible moderating effects of those differing conceptualizations (analogous to Kim et al., 2011). Moreover, from an exploratory perspective, we investigate the modulating effects of the severity of dysfunctional parenting (severe vs. less severe) and the study design (cross‐sectional vs. other) on the associations between parent‐child relationship characteristics and child guilt and shame.

## Objectives

To the best of our knowledge, there is no systematic synthesis of the associations between parent‐child relationship characteristics and child shame and guilt, with the exception of a few narrative and older reviews (Barrett, 1998; Donenberg & Weisz, 1998; Mills, 2005; Reimer, 1996). Thus, the main goal of this meta‐analytic systematic review was to synthesize the available quantitative data in order to (1) determine whether characteristics of the parent‐child relationship are associated with child nonpathological shame and guilt and (2) examine potential sample‐ and study‐level moderators of these associations. We tested the meta‐analytic and meta‐regressive analyses in a confirmatory way if not stated otherwise.

## METHOD

### Registration and protocol

This study was preregistered in a time‐stamped PRISMA‐P study protocol (Moher et al., 2015) on April 26, 2021 (https://osf.io/fnsxj), with protocol amendments on December 19, 2022 (https://osf.io/mdq43) and December 18, 2023 (https://osf.io/6cjxm). The PRISMA‐P checklist (Shamseer et al., 2015) for study protocols and the PRISMA 2020 checklist (Page et al., 2021) are displayed in ESM2 Tables S1 and ESM2 Tables S20.

### Eligibility criteria

Eligibility criteria according to the PICOS framework (Methley et al., 2014) are reported in Table 1. We initially set the mean age of eligible samples at ≤18 years (youth samples) but amended this criterion to include adult samples (i.e., participants aged >18 years; adults reporting on their parent‐child relationship retrospectively together with current reports of shame/guilt) in our analysis. To preserve our focus on general, nonpathological shame and guilt in children and adolescents, we did not examine either specific forms of guilt and shame (e.g., body shame, stigmatization) or their occurrence within clinical phenomena (e.g., callous‐unemotional traits, shame and guilt in mental disorders), and we excluded samples in which the majority of participants exhibited psychopathology. We further specified the inclusion of qualitative studies in our study protocol, but as only one qualitative study was retained after applying all eligibility criteria, we present this study only ESM1 in Data S1.

**TABLE 1:** Inclusion and exclusion criteria according to the PICOS framework.

Criterion	Inclusion	Exclusion
Population	(1) Youth samples: participant mean or median age ≤18 years, (2) adult samples: adults reporting on their parent–child relationship retrospectively, when they were ≤18 years (along with current shame/guilt), or (3) parent reports about the index child ≤18 years	Clinical samples (index child or parent; if >50% of a sample meet the clinical criteria^a^)
		Growing up outside of a stable and long‐term family home
Intervention or exposure	Indicators of the parent–child relationship^a^	Sexual abuse
		Other family relationships (e.g., with siblings, grandparents)
Controls	n.a.	n.a.
Outcome	Quantitative measure or qualitative data of child shame and/or guilt (state or trait)^a^	Specific guilt or shame (e.g., body shame)^a^
		Shame and guilt as part of clinical phenomena (including physical diseases) or stigmatized identity or lack of guilt as a component of callous‐unemotional traits
	For quantitative reports: data to calculate a correlational effect size (Pearson's *r*)	No explicit differentiation between shame and guilt
Study type	Empirical qualitative, quantitative, or mixed‐methods records (published or unpublished)	Nonempirical records (e.g., blog posts, newspaper articles, interviews, audiovisual material collected from web‐based sources)
	English or German publication language	Research syntheses (e.g., systematic reviews and meta‐analyses)
		Purely theoretical or conceptual contributions
		Case studies

^a^ Exact definitions, criteria, and indicators are reported in the preregistered protocol and its amendments.

### Information sources and search strategy

In our systematic literature search, we followed a multitiered search strategy, using preregistered search strings composed of English and German keywords for (a) parents, (b) children and adolescents, (c) shame and guilt, and (d) the parent‐child relationship. On July 22–29, 2021, we searched several large databases with a broad coverage of psychological literature (PSYNDEX, PsycINFO, PubMed, Scopus, and Web of Science [Core Collection]). To gain broader access to grey literature, we further searched Google scholar (first 200 results; sorted by relevance). We conducted a backward search of the included studies in January 2023 and updated our initial search on August 2, 2023 (Web of Science [Core Collection] only). The search strings for each database are displayed in ESM2 Table S2.

### Selection process

Deduplication of the initial search results was performed by the help of the *Deduplicator* (Forbes et al., 2024). After deduplication, the first author and the third author each screened 50% of the records for eligibility based on titles and abstracts (see in ESM2 Table S3). If eligibility was unclear, records were retained for the full‐text assessments. Inter‐rater agreement on study inclusion was assessed in a random subsample of 100 records, yielding a substantial agreement rate of 88% (Cohen's *κ* = .75). Remaining records after the screening stage were assessed in full texts independently by both coders, with a substantial agreement rate of 76% (*κ* = .72). The screening of the updated search results was performed independently by the first author and fourth author, with an inclusion agreement rate of 99% (*κ* = .67).

### Data collection process

The codebook with coding information of all extracted data items is shown in ESM2 Table S4. For the coding procedures, we calculated inter‐rater reliability between the two coders of the initial and updated search on selected variables (see ESM2 Table S5). The mean agreement rate was 84%, with one outlier of 25% (*κ* = .20) but all others between 50% and 100% (Landis & Koch, 1977). All discrepancies were resolved before data analysis.

### Data items

#### Study and sample characteristics

We extracted information on the authors, year of publication, country and year of data collection, study type, study design, and publication language. We further extracted the number of samples per study and their sample sizes, participants' mean age, gender (percentage of female participants), ethnicity, annual household income, and family type.

#### Parent–child relationship

We extracted the indicator of the parent‐child relationship, measurement method, instrument, rater, and the index parent. PCR indicators were classified as either positive (PPCR) or dysfunctional (DPCR) based on a recent meta‐analysis on the PCR (Boele et al., 2019). As outlined in our protocol, we also used broader variables of family functioning and the family system as indicators of the PCR (e.g., cohesion). We further rated whether the assessed dimension of dysfunctional parenting behavior was particularly severe (e.g., abuse, corporal punishment, maltreatment, neglect).

#### Shame and guilt

We extracted the measurement method, instrument, rater, and level (state/trait) of shame and guilt. To define shame and guilt, we used the distinction between self‐attributions (shame) and action‐attributions (guilt; Lewis, 1971). We further distinguished maladaptive from adaptive guilt (“mguilt” and “aguilt”), as the integration of both forms of guilt into one construct would have blurred analyses. An overview of the shame and guilt indicators used in this study, the underlying constructs, and their adaptiveness ratings based on the review by Kim et al. (2011) is displayed in ESM2 Table S6.

#### Effect measure

We extracted the correlation coefficient (Pearson's *r*) between the PCR indicators and child shame or guilt as well as the analytic sample size. For the analysis, we transformed Pearson's *r* into Fisher's *z* following standard recommendations and formulae (Cooper et al., 2019), and for the purpose of presentation, we then transformed summary effect size point estimates and confidence intervals back. Correlations that were only reported as nonsignificant were coded as zero (5 out of 633). Missing information on effect sizes or moderators was either recalculated from data within the study, newly calculated from open data, or obtained from the original authors. Effect sizes with missing data on moderators were omitted from meta‐regressions. Effect size was classified as small, medium, or large with lower thresholds of *r* = .10, *r* = .30, or *r* = .50, respectively (Cohen, 1992).

### Study risk of bias assessment

Study quality was rated using the Mixed Methods Appraisal Tool (MMAT; Hong et al., 2018), a seven‐item tool designed to rate the quality of primary studies with different designs (e.g., qualitative, quantitative including RCT, or mixed‐methods studies) included in a systematic review. We report the ratings of the MMAT in ESM2 Table S7 and discuss the relevant findings in ESM1 Data S1.

### Synthesis of results

Data and annotated R code for this meta‐analysis are openly accessible at https://osf.io/h9ge5/.

#### Meta‐analysis

In total, we conducted six meta‐analyses on the following associations: (1) PPCR × shame, (2) DPCR × shame, (3) PPCR × aguilt, (4) DPCR × aguilt, (5) PPCR × mguilt, and (6) DPCR × mguilt. Three‐level meta‐analyses with robust variance estimation (RVE) were conducted to account for nesting of effect sizes within samples (Hedges et al., 2010; Pustejovsky & Tipton, 2022). Specifically, we applied the correlated and hierarchical effects (CHE) model as proposed by Pustejovsky and Tipton (2022), which assumes a single, known correlation (*ρ*) between effect size pairs from the same study (across all studies) and allows for estimation of within‐ and between‐study heterogeneity (for a more detailed rationale, see ESM1 Data S1). We assumed *ρ* = .60 with additional sensitivity analyses using *ρ* = .20, *ρ* = .40, and *ρ* = .80. Restricted maximum likelihood estimation was used to estimate the model parameters, and we further applied the CR2 correction to adjust for small samples (Pustejovsky & Tipton, 2018).

#### Homogeneity of effect sizes and meta‐regression

We report the between‐sample variance (𝜏^2^) and the within‐sample variance (𝜔^2^) heterogeneity estimate as well as Cochran's *Q*‐statistic and test for heterogeneity. We further calculated the matrix‐based *I* ^2^‐statistic (matrix method; overall, within, and between studies; Nakagawa et al., 2020), rated as low (25%), moderate (50%), or substantial (75%; Higgins & Thompson, 2002). Additionally, we calculated 95% prediction intervals, that is, intervals within which the true effect is expected for 95% of future, similar studies (Cooper et al., 2019; IntHout et al., 2016). We adapted the calculation of the 95% prediction interval in *metafor* (Viechtbauer, 2010) based on robust standard errors.

We ran a series of meta‐regression models with a range of variables as moderators, separately for each moderator and meta‐analysis. As outlined above, these included characteristics of the study design (year, methods, etc.), study quality (MMAT), study variables (rater, operationalization, parenting severity, etc.), and sample (gender, age, geographical region, ethnicity, etc.). After the preregistration, we identified other potential confounders to be tested (see protocol amendments). Following the inclusion of adult samples, we investigated “age‐group” (youth vs. adults) as a dichotomous moderator. Although most of the adult samples reported a rather low average age of 19–29 years (*M*_age_ = 22.47, *SD*_age_ = 3.74), which corresponds to emerging adulthood (Arnett et al., 2014), they mostly also indicated a wide age range of up to 51 years (*M*_range_ = 23.4 years) within the original samples. Thus, we deemed age an unsuitable candidate variable to be considered as a continuous moderator within the adult samples for the following two reasons. First, the age mean might be biased by the inclusion of younger and older adults in the samples, which have a wide age range. Second, the retrospective reports of the PCR in these samples include both very short and long periods of time for the younger and older adults, respectively. Due to the potentially confounding effects of the different retrospective time periods covered and since we consider the average age to be an inaccurate statistical measure in the absence of other distributional estimates in the adult samples, we examined age as a continuous moderator only in the youth samples.

We compared broader variables of family functioning and the family system as indicators of the PCR (e.g., cohesion) in addition to those in the narrower sense. Samples that were primarily considered as clinical were excluded, but in the remaining samples, we extracted whether any psychopathology was reported throughout the sample and investigated its potential confound. A full list of moderators including operationalization and coding scheme is displayed in ESM2 Table S8.

We assumed the same CHE model structure as for our intercept‐only models (i.e., constant sampling correlation *ρ* = .60, effect sizes nested within samples) with the inclusion of the respective moderator (Pustejovsky & Tipton, 2022). Statistical significance of moderators was assessed using the Wald test with CR2 correction (Pustejovsky, 2020).

#### Publication bias and decline effects

To date, the assessment of publication bias in meta‐analyses with dependent data is still an unresolved issue, as only regression‐based methods to detect small study effects—such as three‐level/RVE variants of Egger's test—have been found to perform favorably in simulation studies (Rodgers & Pustejovsky, 2021). This runs counter to current recommendations favoring method triangulation (Siegel et al., 2022), but other families of methods are not yet suited to handle effect size dependency (Rodgers & Pustejovsky, 2021). Thus, we applied three variants of Egger's test (Rodgers & Pustejovsky, 2021), supplemented by visual inspections of funnel plots. Publication status (unpublished vs. published) was further investigated in meta‐regressions. Additionally, we assessed time trends in the effect sizes for decline effects by including publication year as a moderator and by visual inspection of the effect sizes plotted against time (Pietschnig et al., 2019).

### Statement on the use of generative artificial intelligence

During preparation of this manuscript, ChatGPT (versions 3.5 and 4; OpenAI, 2024) was used to streamline the coding process in R. Prompts included varieties of statements such as: “Please generate a code that generates a table incorporating the results of an rma.mv model.” ChatGPT and DeepL (DeepL, 2024) were further used in rephrasing words or sentences of written manuscript text. The first author incorporated these suggestions as seen fit and, together with the other authors, takes full responsibility for the content of this manuscript. Crucially, no new text segments, ideas, references, or statistical analyses were generated with the help of ChatGPT, any other large language model, or any other artificial intelligence assistance.

## RESULTS

### Study selection

**FIGURE 1:**
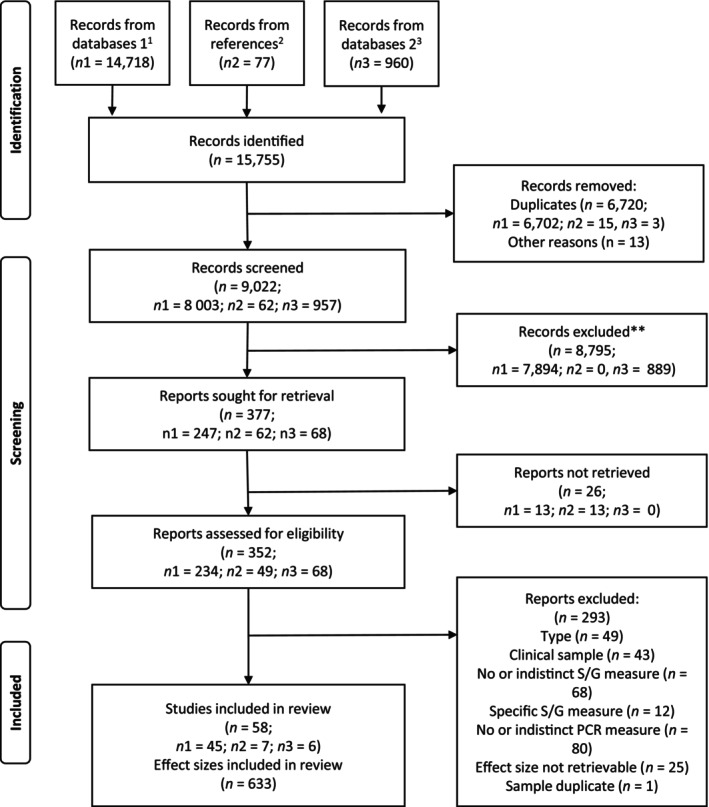
Flow diagram. ^1^Initial search; ^2^Backward search of the records identified in the initial search; ^3^Updated search. **Records excluded based on the preregistered screening criteria.

The PRISMA flow diagram depicting the study search and selection process from the first search to the final analysis is presented in Figure 1. The majority of included studies (90.8%) provided multiple effect sizes and 12.1% drew results from multiple samples. In our model, we thus applied the structure of effect sizes nested within samples instead of studies to avoid aggregating effect sizes on the study level. A narrative description of the retention of samples and effect sizes can be found in ESM1 Data S1.

### Study characteristics and study risk of bias

Characteristics of the included studies are displayed in Table 2. The study quality ratings of the included studies according to the MMAT are displayed in ESM2 Table S7.

**TABLE 2:** Study descriptive statistics of included studies.

ID	Author(s)	Year	ctry	*n*	*N*	Age	Fem (%)	*k*	PCR‐I	PCR‐D	PCR‐P	PCR‐R	EMO‐E	EMO‐M	EMO‐R	*t*
1	Abell & Gecas	1997	US	1	270	20.70	61.5	32	Parenting (affective/coercive/inductive control, support)	pos, dys	M, F	S, G	Shame, aguilt	TOSCA‐3	C	1
2	Ackland	2000	CA	1	49	2.03	49	1	Parenting (authoritarian)	dys	P	P	Aguilt	Behavioral coding	R	0
3	Ahmed	1999	AU	1	978	10.90	53	6	Stigmatized shaming, nonstigmatized shaming, family disharmony	dys	P	P	Shame, aguilt	TOSCA‐C	C	1
4	Alessandri & Lewis	1993	US	1	30	3.04	46	6	Parental evaluations (positive, negative)	pos, dys	P	R	Shame	Behavioral coding	R	0
5	Ashy et al.	2020	US	2	200, 239	27.00	0, 100	16	Parental aggression (physical, psychological)	dys	M, F	C	Shame, mguilt	PFQ2	C	1
6	Assor & Tal	2012	IL	1	153	16.50	51	3	Parental conditional regard (positive, negative), parental control	dys	P	C	Shame	PFAI	C	1
7	Bahtiyar & Gençöz	2023	TR	1	544	26.50	75	6	Parenting (overprotection, rejection, warmth)	pos, dys	M	C	Shame, aguilt	TOSCA‐3	C	1
8	Belsky et al.	1997	US	1	110	3.08	0	4	Parenting (negative, positive)	pos, dys	F, M	R	Shame	Behavioral coding	R	0
9	Bennett et al.	2005	US	1	177	5.00	47	2	Physical abuse, neglect	dys	P	R	Shame	Behavioral coding	R	0
10	Brown	1978	US	2	42, 72	12.50	0, 100	12	Parenting (inductive, love withdrawal, power assertive)	dys	P	C	Aguilt	Story task	R	1
11	Caplovitz Barrett	2005	US	1	35	1.42	49	3	Gentle enforcement of standards, reactions to adhering to standards, hostility	pos, dys	P	R	Aguilt	Behavioral coding (low guilt)	R	0
12	Chandler‐Holtz	1999	US	2	259	16.60	58	72	Parenting (child centeredness, control through guilt, harsh discipline, hindering individuation, inconsistency, instilling persistent anxiety, overprotection, permissiveness, psychological intrusiveness, rejection, warmth, withdraw relations)	pos, dys	M, F	C	Shame, aguilt, mguilt	TINSA	C	1
13	Claesson & Sohlberg	2002	SE	2	62, 122	n.a.	0, 58	6	Disrupted attachment (attack, blame, ignore)	dys	M	C	Shame	ISS	C	1
14	Clifton	1995	US	1	218	19.70	72	6	Parenting (ambivalent/inconsistent, cold/rejecting, warm/responsive)	pos, dys	M, F	C	Shame	YSQ	C	1
15	Coates & Messman‐Moore	2014	US	1	771	18.80	100	5	Parenting (corrupting, demanding/rigid, emotional nonresponse, spurning/terrorizing), maltreatment	dys	P	C	Shame	YSQ‐S	C	1
16	Cornell & Frick	2007	US	1	87	4.39	52.9	3	Parenting (corporal punishment, inconsistent discipline, positive reinforcement)	pos, dys	P	P	Aguilt	My child	P	1
17	Crane et al.	2020	US	1	322	15.20	n.a.	2	Family implicit rules (facilitating, constraining)	pos, dys	P	P	Shame	ISS	C	1
18	DeRobertis	2000	US	1	3	20.60	67	0	Psychological maltreatment	dys	P	C	Shame	Interview	C	1
19	Donatelli et al.	2007	US	1	43	15.30	53	4	Parenting (maladaptive guilt induction)	dys	M	C	Shame, mguilt	PFQ2	C	1
20	Donohue et al.	2020	US	1	4485	10.00	47	2	Parenting (rejection)	dys	P	C, P	Mguilt	KSADS‐5 (single item)	C, P	1
21	Dos Santos et al.	2020	PT	1	69	8.80	43	16	Parenting (behavior‐focused, humiliation, love withdrawal, person‐focused, power assertion, victim‐focused induction)	pos, dys	P	P	Shame, aguilt	TOSCA‐C	C	1
22	Dyer et al.	2022	US	1	617	13.10	47	12	Family flexibility, verbal hostility	pos, dys	F, M, P	C	Shame	ISS	C	1
23	Essler & Paulus	2022	DE	1	220	1.12	50	11	Parental reactions to transgression, tendency to react with negative emotions or verbal direction, authoritarian tendencies	pos, dys	M	R	Aguilt	Self‐developed question	M, R	0
24	Gilbert et al.	1996	US	1	80	24.60	100	8	Parenting (care, favoritism, overprotection, put‐down)	pos, dys	M, F	C	Shame	ISS	C	1
25	Gülseven et al.	2022	TR	1	204	18.70	62.5	4	Parenting (psychological control, support)	pos, dys	P	C	Shame, aguilt	TOSCA‐3	C	1
26	Han & Kim	2012	KR	1	1185	11.50	48.5	2	Parenting (rejection)	dys	M, F	C	Shame	SSGS	C	0
27	Harper & Arias	2004	US	2	120, 174	19.00	0, 100	2	Psychological maltreatment	dys	P	C	Shame	TOSCA‐3	C	1
28	Harris & Curtin	2002	US	1	194	19.30	60	2	Parenting (care, overprotection)	pos, dys	P	C	Shame	YSQ	C	1
29	Hoglund	1996		2	504	22.80	0, 100	9	Emotional abusiveness	dys	M, F, P	C	Shame	ASGS, TOSCA	C	1
30	Jones et al.	2006	UK	1	50	30.80	100	2	Parenting (rejection, warmth)	pos	F	C	Shame	YSQ‐S	C	1
31	Jurkowitz	1996	US	1	91	15.60	52	12	Communication (open, problem)	pos, dys	P	C, P	Shame	ISS, PFQ2	C	1
32	Karos	2007	CA	1	66	7.80	46	120	Parenting (authoritative, authoritarian, permissive, attachment, conditional approval, disgust, humiliation, love withdrawal, mother self‐focus, positive/negative child focus, power assertiveness, self‐reliance, supportive/unsupportive emotion response)	pos, dys	M	M	Shame, aguilt, mguilt	SCEMAS, My child	C, M	1
33	Kelley et al.	2000	US	1	75	3.00	47	7	Parental behavior (corrective/positive/negative feedback, gentle guidance, intrusive control)	pos, dys	M	R	Shame	Behavioral coding	R	0
34	Kochanska et al.	2005	US	1	99	2.73	53	1	Parenting (power assertion)	dys	M	R	Aguilt	Story task	R	1
35	Kochanska et al.	1996	US	1	103, 99	2.74, 3.83	49	4	Parenting (power assertion)	dys	M	R	Aguilt	Behavioral coding	R	0
36	Lutwak & Ferrari	1997	US	1	404	20.40	65.4	4	Parenting (care, protection/control)	pos, dys	M, F	C	Shame	ASGS	C	1
37	Luu	2002	US	1	297	20.90	68	24	Parenting (affectionate, guilt engendering, overcontrol, performance‐oriented, rejection, stimulating, tolerant)	pos, dys	P	C	Shame, aguilt	ISS, TOSCA‐3	C	1
38	Marici et al.	2023	RO	1	220	17.10	68.7	4	Parenting (rejection)	dys	F, M	C	Shame, mguilt	ESS, Guilt Inventory	C	1
39	Matos & Pinto‐Gouveia	2014	PT	1	230	34.20	70	2	Shame traumatic memory & shame memory centrality	dys	P	C	Shame	ISS	C	1
40	McGinniss	1997	US	1	59	19.80	74	28	Parenting (acceptance, active involvement, achievement/hostile/lax/punitive/strict control, cognitive competence/curiosity/independence, conformity, equalitarianism, neglect, punishment, rejection)	pos, dys	M, F	C	Shame	TOSCA‐3	C	1
41	Meesters et al.	2017	NL	1	477	13.90	53	28	Parenting (rejection, warmth)	pos, dys	M, F	C	Shame, aguilt, mguilt	SCEMAS	C	1
42	Messman‐Moore & Coates	2007	US	1	382	19.30	100	5	Parenting (control, warmth), psychological maltreatment	pos, dys	M, F	C	Shame	YSQ	C	1
43	Mills	2003	CA	1	104	3.80	100	6	Parenting (authoritarian)	dys	M, F	P	Shame	Behavioral coding	R	0
44	Mills et al.	2010	CA	2	97, 128	4.14, 5.89, 8.15	0, 100	24	Parental shaming/authoritarian parenting	dys	M, F	M, F	Shame	Behavioral coding, TOSCA‐C	R, C	0, 1
45	Mintz et al.	2017	US	1	213	28.10	53.7	16	Parenting (bonding, discipline, education, negativity, positive, responsivity, sensitivity, welfare/protection)	pos, dys	P	C	Shame, aguilt	TOSCA‐3	C	1
46	Murray et al.	2000	UK	1	139	21.00	100	8	Care, protection/control	pos, dys	M, F	C	Shame	ISS, TOSCA‐3	C	1
47	Pulakos	1996	US	1	152	21.50	67.8	8	Family system (cohesion, control, expressiveness, organization)	pos, dys	P	C	Shame	TOSCA‐3	C	1
48	Rote et al.	2021	US	1	123	14.00	54	24	Parenting (hostility, lecture, warmth, whining)	pos, dys	M	C, M, R	Shame, aguilt	Self‐reflection	C	1
49	Sedighimornani et al.	2021	UK	1	240	27.80	80.1	3	Parenting (care, expectations/perfectionism, negative attitude toward emotions)	pos, dys	P	C	Shame	ESS	C	1
50	Smiley et al.	2020	US	1	98	11.60	54	2	Parental conditional regard (positive, negative)	dys	P	M	Shame	Self‐reflection	R	1
51	Stuewig & McCloskey	2005	US	1	363	9.30, 15.00	52	6	Parenting (harsh, rejection, warmth)	pos, dys	P	C	Shame, aguilt	TOSCA‐C	C	1
52	van Eickels et al.	2022	AT	1	526	15.70	76.4	2	Family cohesion	pos	P	C	Shame, aguilt	TOSCA‐A	C	1
53	Veneziani et al.	2023	IT	1	710	23.30	75.1	12	Parenting (indifference, overcontrol), abuse	dys	M, F	C	Shame	GASP	C	1
54	Walisever	1995	US	1	20	20.00	80	11	Attachment (secure, avoidant, ambivalent), parenting (coercion/guilt/rational discipline, independence encouragement, love withdrawal)	pos, dys	M, F, P	C	Mguilt	PFQ2 (parent‐specific)	C	1
55	Walter & Burnaford	2006	US	1	175	15.80	57.7	4	Closeness	pos	F, M	C	Shame, aguilt	TOSCA‐A	C	1
56	Webb et al.	2007	US	1	280	20.90	65	6	Emotional neglect, isolation, rejection	dys	P	C	Shame, aguilt	TOSCA‐3	C	1
57	Wells & Jones	2000	US	1	197	21.00	65	2	Parentification	dys	P	C	Shame, aguilt	TOSCA‐3	C	1
58	Wright et al.	2017	US	1	172	19.30	73	1	Warmth	pos	P	C	Shame	TOSCA‐3	C	1

*Note:* Included studies are marked with * in the reference list.

Abbreviations: ctry, country of data collection (ISO 3166); EMO‐E, assessed emotion(s) [shame, adaptive (aguilt) or maladaptive guilt (mguilt)]; EMO‐M, measurement instrument of shame/guilt; EMO‐R, rater of shame/guilt [C, child; F, father; M, mother; P, parents; R, researcher]; fem (%), percentage of female participants; ID, study ID; *k*, number of effect sizes; *n*, number of samples; *N*, sample size; PCR‐D, dimension of assessed indicator(s) of the parent–child relationship [dys, dysfunctional; pos, positive]; PCR‐I, assessed indicator(s) of the parent–child relationship; PCR‐P, index parent for whom the PCR is rated [F, father; M, mother; P, both parents]; PCR‐R, rater of the parent–child relationship [C, child; F, father; M, mother; P, parents; R, researcher]; *t*, trait emotion (1 = yes, 0 = no); year, year of publication.

### Sample descriptive statistics

Participants' mean age across all samples that reported age and including adult samples (weighted by sample sizes) was 15.24 years (SD = 6.62; youth samples only: *M* = 10.93 years; SD = 3.42). The weighted gender distribution throughout the sample, where reported, was 59.0% girls or women, with no study reporting other than binary gender. Data collection mostly took place in North America (75.4%), followed by Europe (18.4%; Western: 16.9%, Eastern: 1.5%), the Middle East (3.1%), Australia/New Zealand (1.5%), and East Asia (1.5%). Ethnicity was reported in 90.9% of the U.S. samples and in no study from geographical regions other than the United States. Where reported, participants' ethnicity across the U.S. samples was 65.0% White, 12.3% Hispanic and Latinx, 10.8% Black, 6.3% mixed race, 5.6% Asian American, 0.3% Native American, and <0.1% undefined or other. The reported mean annual income was 68,381 USD (U.S. studies only). Recruitment was rated as probabilistic in 32.3% of the studies (MMAT2 ratings for quantitative descriptive studies). The detailed sample descriptive statistics, both overall and for each meta‐analytic model separately, are displayed, in part, in Table 3 and, in full, in ESM2 Table S9.

**TABLE 3:** Descriptive statistics per meta‐analysis.

	Full sample	PPCR × shame	DPCR × shame	PPCR × aguilt	DPCR × aguilt	PPCR × mguilt	DPCR × mguilt
Records							
Studies	58	29	46	16	21	5	9
Samples	65	29	52	16	23	5	11
Peer‐reviewed	49	3	42	1	0	1	6
Clinical^a^	7	23	5	12	14	0	2
Effect sizes (*k*)	633^d^	110	264	56	116	22	65
Sample							
*N*	19,144	6747	13,228	3536	4811	913	6256
*n*_youth_	11,834	3303	5941	2400	3198	893	5797
*n*_adults_	7310	3444	7287	1136	1631	20	459
Female participants^b^	11,295	4359	8306	2107	2722	497	3108
Annual income *M* (SD)^c^	68,381 (36,553)	53,516 (23,013)	70,561 (36,814)	28,708 (11,306)	32,702 (11,276)	40,365 (11,906)	39,382 (10,096)
Age							
*M* (SD)	15.24 (6.62)	17.85 (5.81)	17.66 (6.36)	15.20 (6.36)	14.35 (6.51)	14.52 (2.34)	12.30 (4.67)
Min	1.12	3.00	3.00	1.12	1.12	7.80	7.80
Max	34.23	30.78	34.23	28.06	28.06	20.00	27.00
*M*_youth_ (SD)	10.93 (3.42)	13.10 (3.51)	11.94 (3.38)	12.03 (4.88)	10.69 (4.50)	14.39 (2.22)	11.16 (2.38)
*M*_adults_ (SD)	22.47 (3.74)	22.41 (3.44)	22.48 (3.75)	21.89 (3.09)	21.61 (2.63)	20.00 (0.00)	26.69 (1.43)
Geographical region (samples)							
Australia & NZ	1	0	1	0	1	0	0
East Asia	1	0	1	0	0	0	0
Europe (East)	1	0	1	0	0	0	1
Europe (West)	11	6	9	4	3	1	1
Middle East	2	1	2	0	0	0	0
USA & Canada	49	22	38	12	19	4	9

*Note:* Excerpt of ESM2 Table S9. Full sample = all samples across meta‐analyses including the qualitative study. Studies = publications (may pertain multiple samples).

Abbreviations: aguilt/mguilt, adaptive/maladaptive guilt; NZ, New Zealand; PPCR/DPCR, positive/dysfunctional parent–child relationship.

^a^ Samples with up to 50% clinical participants. Samples with >50% clinical representation are excluded.

^b^ Weighted *n* based on the percentage of female participants per sample.

^c^ Annual income in USD calculated exclusively for the U.S. samples.

^d^ The full data set contains 634 rows, including the qualitative study that yielded 0 effect sizes.

### Meta‐analyses

Overall, 633 effect sizes from 64 samples were included in the six meta‐analyses (see Table 4). Orchard plots are depicted in Figures in ESM3 S1–S6. First, regarding shame as outcome, we found a small negative association between PPCR and child shame (*r* = −.12; 95% CI [−.17; −.06]) and a small positive association between DPCR and child shame (*r* = .18; 95% CI [.14; .21]). Second, regarding adaptive guilt as outcome, we found a small positive association between PPCR and child adaptive guilt (*r* = .14; 95% CI [.09 .18]) but no significant association between DPCR and child adaptive guilt (*r* = −.01; 95% CI [−.03; .02]). Third, regarding maladaptive guilt as outcome, there was no significant association between PPCR and child maladaptive guilt (*r* = .01; 95% CI [−.10; .12]), but we found a small positive association between DPCR and child maladaptive guilt (*r* = .15; 95% CI [.09; .22]). As we suspected implausible effect sizes from study ID 54 (*r* > .70), we re‐ran the respective analysis excluding them, which yielded a marginal change (*r* = .02 in both cases, no changes in statistical significance) in the results (PPCR × maladaptive guilt: *r* = .03, 95% CI [−.09; .14], *p* = .493; DPCR × maladaptive guilt: *r* = .13, 95% CI [.07; .19], *p* = .001). Sensitivity analyses using correlations of *ρ* = .20, .40, and .80 between effect sizes within studies yielded only trivial variation in summary effect sizes (*z* = .000–.037; *M* = 0.009; Md = 0.004) and none in significance (see in ESM2 Table S10).

**TABLE 4:** Meta‐analyses, assessment of heterogeneity, and prediction intervals.

	PPCR × shame	DPCR × shame	PPCR × aguilt	DPCR × aguilt	PPCR × mguilt	DPCR × mguilt
Descriptive statistics						
*k*	110	264	56	116	22	65
*n*	29	52	16	23	5	11
*N*	6747	13,228	3536	4811	913	6256
ES						
lo	−0.40	−0.29	−0.46	−0.30	−0.55	−0.20
hi	0.33	0.49	0.35	0.39	0.38	0.87
Year						
Min	1993	1993	1996	1978	1995	1995
Max	2022	2023	2022	2022	2017	2023
Meta‐analysis						
*r*	**−.12**	.**18**	.**14**	−.01	.01	.**15**
*z*	**−.12**	.**18**	.**14**	−.01	.01	.**15**
95% CI						
LL	−0.17	0.14	0.09	−0.03	−0.10	0.09
UL	−0.06	0.21	0.18	0.02	0.12	0.22
*p*	**<.001**	**<.001**	**<.001**	.681	.853	.**001**
Heterogeneity assessment						
*τ* ^2^	.01	.01	.00	.00	.00	.00
*ω* ^2^	.01	.01	.01	.01	.02	.01
*Q*						
*Q*	**570.09**	**1429.42**	**209.18**	**484.30**	**88.24**	**236.52**
df	109	263	55	115	21	64
*p*	**<.001**	**<.001**	**<.001**	**<.001**	**<.001**	**<.001**
*I* ^2^						
Total	88.50	89.39	78.88	80.43	83.81	84.25
Between	42.77	43.56	12.75	0.00	0.00	15.85
Within	45.73	45.83	66.13	80.43	83.81	68.40
95% PI						
LL	−0.39	−0.09	−0.06	−0.21	−0.28	−0.06
UL	0.17	0.42	0.32	0.19	0.30	0.35

*Note:* 95% CI, 95% confidence interval of *r*; 95% PI, 95% prediction interval of *r*; aguilt/mguilt, adaptive/maladaptive guilt; df, degrees of freedom; ES, range of effect sizes in the original studies; hi, highest effect; *I* ^2^, *I* ^2^ statistic for between‐ and within‐study heterogeneity and their total sum; *k*, number of effect sizes; LL, lower level; lo, lowest effect; max, newest publication; min, oldest publication; *N*, independent sample size; *n*, number of samples; PPCR/DPCR, positive/dysfunctional parent–child relationship; *Q*, *Q*‐statistic; *r*, Pearson's correlation coefficient *r* (recalculated from *z*); UL, upper level; year, year of publication; *z,* Fisher's *z*; *τ* ^2^, tau^2^, between‐sample variance; *ω* ^2^, omega^2^, within‐sample variance. Significant values are in bold.

### Meta‐regression

We found substantial within‐ and between‐study heterogeneity in all meta‐analyses, as reflected by *I* ^*2*^ and *Q* (see Table 4). The 95% prediction interval always included zero and varied in size from .40 to .58 between meta‐analyses. A total of 27 moderators were tested (see in ESM2 Table S8). The results for each meta‐analytic model are displayed in ESM2 Tables S11–S16. In total, 11 of the 27 moderators showed 18 significant effects. However, 10 of these effects compared only one study against the rest of the sample. As we cannot rule out confounding effects based on study‐specific characteristics, we refrain from an extensive discussion of these results and instead focus on the overall significant results with a minimum of two studies in the same moderator category. We further elaborate on the nonsignificant findings in the discussion.

The association between PPCR and shame was significantly stronger in samples with a higher proportion of females and a higher annual income (U.S. samples), and spuriously stronger at *p* = .051 for studies assessing broader family‐level indicators compared to narrower PCR indicators. The association between DPCR and shame was stronger in cross‐sectional studies and when PCR measures were reported by the child compared to parents or other raters. The association between PPCR and adaptive guilt was stronger in studies that conceptualized guilt as internalized than those that applied other measures. The association of DPCR and maladaptive guilt was stronger in studies that conceptualized maladaptive guilt as internalized compared to maladaptive guilt‐proneness. Our planned moderator analyses concerning age included the comparison between youth and adult samples as well as its testing as a linear predictor in the youth samples only. Upon the investigation of weighted scatterplots (Figures in ESM3 S7–S12), we further included a quadratic term for age in the moderator analyses within the youth samples in an exploratory fashion. We found significant quadratic effects of age for PPCR and shame (inverse U‐shaped) and for DPCR and shame (U‐shaped; see in ESM2 Tables S11 and S12). The main analyses and age‐related meta‐regressions further proved to be stable regardless of inclusion versus exclusion of samples with *M*_age_ < 24 months (ESM2 Tables S17 and S18).

### Publication bias

Publication status and year of publication did not moderate the effects. We further found no indication of publication bias based on Egger's test and funnel plots (ESM2 Table S19 and in ESM3 Figures S13–S18), except for the association between DPCR and maladaptive guilt. After the removal of study ID 54 (analogously to the meta‐analysis), the funnel plot appeared more symmetric (ESM3 Figure S19) but Egger's test remained significant for the RVE model. The absence of cohort or decline effects within the meta‐regressions was supported by visual displays of the effect sizes over time (ESM3 Figures S20–S25) in addition to the nonsignificant meta‐regressions with publication year as a predictor.

## DISCUSSION

Our analyses revealed that indicators of a dysfunctional parent‐child relationship are associated with feelings of shame and maladaptive guilt in children and adolescents, but not with adaptive guilt. Conversely, positive parent‐child relationship characteristics appear to create a supportive context for adaptive guilt and serve as a protective factor against shame but not against maladaptive guilt. We based our certainty in these results on our meta‐analytical models, including the gradual testing of different variance matrices, and on a thorough examination of moderators and sources of bias. In theory, children's social‐emotional development is largely shaped by their relational experiences with their primary caregivers (Grusec, 2011). The present findings empirically support this notion through a rigorous meta‐analytical approach.

The small effects revealed in our meta‐analysis correspond to those reported in previous meta‐analyses regarding the association of parenting with child psychopathology (Pinquart, 2017) and child negative emotionality (Cooke et al., 2019; Paulussen‐Hoogeboom et al., 2007). It is important to note that there are no deterministic, linear relations between parenting and child outcomes (Sroufe, 1997). The development of shame and guilt is influenced by a complex interaction of a myriad of factors located within the individual, such as genetics and temperament, and in the wider external environment, such as peer influence, bullying experiences, or traumatic events (Sedighimornani et al., 2021; Szentágotai‐Tătar et al., 2015). Examining potential mediating mechanisms from parental behaviors to child shame and guilt via self‐attributions and internal working models (Mills, 2005) and considering possible bidirectional as well as prospective effects between parent and child behavior in future research might improve the understanding of intra‐family processes and the impact of self‐conscious emotions on family relationships. Given that most studies in this review were cross‐sectional (88.3%), we cannot determine causality or the direction of effects. A bidirectional association is likely considering that children actively interact with their environment from infancy onward (Kiff et al., 2011), and their shame and guilt are associated with differential behavioral outcomes, such as appeasement or aggression (Dempsey, 2017; Elison et al., 2006).

Substantial heterogeneity is common in psychological meta‐analyses, and the within‐ and between‐study variance was thus—unsurprisingly–high across our sample. We attribute this primarily to the fact that we accumulated evidence on a wide range of shame and guilt measures as well as indicators of the parent‐child relationship. Although we tried to reduce the conceptual heterogeneity by theoretically meaningful classifications (e.g., adaptive vs. maladaptive guilt; PPCR vs. DPCR characteristics), slight conceptual blurring could remain. When exploring possible sources of heterogeneity, we found significant moderating variables specific to samples and methods in meta‐regressions. There were significant moderators in analyses that compared only one sample against the rest within the respective analysis. We advise a cautious interpretation of these effects given they might be confounded with specific characteristics of a single study and have not shown a robust, generalizable pattern. Therefore, we only discuss those that compared effect sizes from at least two studies to the rest of the sample to ensure robustness.

On the sample level, we found that a higher percentage of female participants was associated with a more negative association between PPCR and shame (*b* = −.003), signifying a .03 increase in effect size per 10% increase in female participants in the sample. Thus, gender may exert a differential influence depending on parenting behavior and outcome, in such that associations might be stronger for girls than for boys. Furthermore, we found that the association between PPCR and shame was more negative in samples with a higher annual income in USD (analyzed within the U.S. samples only for homogeneity). Here, one might suspect a protective effect of social privilege or confounding effects of stigma (Jo, 2013). The association between PPCR and shame showed a trend toward stronger effects for broader family system variables (e.g., cohesion) compared to genuine parent‐child relationship variables. This might point to confounding effects of a more positive family climate or to compensatory effects of nonparent relationships within the family on shame but not on any of the other emotions examined. We found no significant relation between DPCR and shame in the longitudinal study samples. This raises the question of the most appropriate time intervals to measure the associations between parenting and child emotionality but also suggests that within‐person and within‐dyad trajectories might differ from the analyzed between‐person effects (Lougheed & Keskin, 2021). Furthermore, the correlation between DPCR and maladaptive guilt was stronger in studies in which maladaptive guilt was assessed as internalized guilt rather than as maladaptive guilt‐proneness. The correlation between PPCR and adaptive guilt was strongly negative in one study that assessed behavioral guilt and thus was significantly different from the overall positive coefficients found when guilt was assessed other than behavioral. It has to be noted that this effect size was recoded and was the only effect size in this category, limiting its interpretability. Still, samples that used a measure of internalized guilt yielded significantly stronger effects compared to those that used a measure other than behavioral guilt or guilt‐proneness. These findings underline the importance of examining the conceptual and theoretical differences between core measures across studies in this field, which can only be revealed through meta‐analytic efforts (Lear et al., 2022). Lastly, the significantly stronger association between DPCR and shame when the child reported the parent‐child relationship (compared to parents or other raters) may indicate differences in either the measure or the perception of the parent‐child relationship.

Although we have focused on the results of the significant meta‐regressions in the discussion, the nonsignificant results deserve some attention. Our summary effects showed considerable stability across most moderator variables tested. This includes the gender of index parent, severity of dysfunctional parenting, inclusion of clinical samples, or represented ethnicity. Further, we found only single‐study moderating effects of the state or trait level of emotions, the age group (youth vs. adult samples), or the geographical region. The stability of the results may indicate a certain universality of the found effects, but we refrain from over‐interpreting the nonsignificant meta‐regressions since the statistical power of our meta‐regressive analyses might be limited. Therefore, we encourage future research to address these issues in more detail to reach more robust conclusions.

Perhaps, most important from a developmental perspective is that we found no differences between the youth and adult samples and no linear age effects within the youth samples either. We, however, visually identified and statistically found quadratic effects of age in the youth samples for both meta‐analyses of shame. These results suggest that the association between PPCR/DPCR and shame appears to be most pronounced in toddlerhood and adolescence but is diminished in middle childhood. In view of the exploratory nature of this analysis, we can only speculate about the reasons for this finding.

Toddlerhood is an especially sensitive phase for attachment, which is strongly predictive of the child's emotional make‐up (Cooke et al., 2019). Further, the development of self‐conscious emotions emerges during this developmental period and is therefore particularly malleable to outer influences during this time (Thompson, 2019). A child's mental capacity for shame might further enhance in middle childhood through cognitive maturation (Misailidi, 2018), however not (yet) as intertwined with the self‐concept as in adolescence (Reimer, 1996). The general trajectories of shame and guilt over adolescence are less studied, but there is evidence for a decrease in shame and an increase in guilt from adolescence into middle adulthood (Orth et al., 2010). One possible explanation for this is the “maturity principle” that claims a general increase of adaptive and decrease of maladaptive emotions over the lifespan (Orth et al., 2010). Reimer (1996) argues that shame might also increase in adolescence due to cognitive maturation and heightened sensibility to social cues, as well as social and bodily challenges. Further, social emotions might intensify in adolescence, a period of both individuation and autonomy seeking as well as attachment needs. A warm and supportive family environment likely helps adolescents through this challenging phase of identity formation. Our data support Reimer's (1996) notion that PPCR characteristics are associated with a decreasing trajectory of shame in adolescence, while the opposite was found for dysfunctional parenting. The accentuation of the relation between shame and the parent‐child relationship may thus be driven by a renegotiation and balancing of young people's needs within the parent‐child relationship rather than a normative increase or decrease (Koepke & Denissen, 2012).

The use of mostly parental or researcher report in studies examining this age group could further inflate effects. Conversely, due to cognitive maturation, the reliability of self‐report measurements might increase again in adolescence (Keefer, 2015). Associations might also inherently strengthen at the rise of social comparisons in adolescence and the formation of new relationships based on the parent‐child relationship (Brown & Bakken, 2011). However, due to the lack of longitudinal studies, this explanation remains tentative. We did not find a significant quadratic association for adaptive or maladaptive guilt, but regardless of significance, the effect sizes of the quadratic terms were similar in size across meta‐analyses. We suspect that statistical power played a role in the significance of these results.

The effects in toddler samples are further to be discussed. From a developmental psychological perspective, it is assumed that children can experience shame and guilt from around 24 months (Lewis, 2008). Since the main analyses and age‐related meta‐regressions showed to be robust against inclusion versus exclusion of two samples with a mean age below 2 years, we retained these samples in the final dataset.

With the exception of single‐study effects related to measurement reporting in two analyses, no study quality indicator exhibited significant effects across meta‐analyses (see Data S1). We therefore conclude that our main findings are quite robust to potentially biased methods within the primary studies according to the criteria of the MMAT (Hong et al., 2018). Overall, we also found no indication of publication bias, except for the association of DPCR and maladaptive guilt, which might be partly attributable to our effect size of interest (Pearson's *r*) being commonly reported while not being the main outcome of most studies.

In sum, the results of our meta‐analysis support the notion that the parent‐child relationship is associated with child shame and guilt. However, another question that has not yet been conclusively answered and was also not investigated in the present study is *why* this association exists. As briefly outlined in the introduction, different psychological theories offer explanations for the importance of the parent‐child relationship in children's development of shame and guilt. As our review does not favor one theory over another and a theoretical integration was not our goal, we conclude that the parent‐child relationship plays an important role, albeit modest in terms of effect sizes, in the development of child shame and guilt, and advocate for further comprehensive theoretical work in this field.

### Practical implications

The finding that indicators of the parent‐child relationship are differentially related to child shame and guilt bears important practical implications. The positive influence of a supportive parent‐child relationship underscores its significance as a nurturing environment to foster children's adaptive emotional responses. Conversely, the impact of dysfunctional parenting on negative emotional outcomes in children emphasizes the need to promote healthier parent–child dynamics. As briefly mentioned in the introduction, shame and maladaptive guilt, in contrast to adaptive guilt, play an important role in the development and maintenance of mental disorders (Cândea, & Szentagotai‐Tatar, 2013; Bottera et al., 2020; Kim et al., 2011; Muris & Meesters, 2014). Shame‐proneness and maladaptive guilt have further been found to mediate pathways from parental behavior to psychopathological symptoms in cross‐sectional studies (e.g., van Eickels et al., 2022; Veneziani et al., 2023), while there is a general scarcity of longitudinal research to establish methodologically sound mediation effects. Moreover, it should be noted that feeling ashamed or guilty when transgressing social norms or hurting others is important for prosocial behavior, and thus a lack of shame and guilt is considered dysfunctional from a developmental psychopathology perspective (Olthof, 2012). In this vein, our meta‐analytic results might shed light on a potential mechanism mediating the effects of parenting behavior on child outcomes in subclinical and clinical research, although the current review only focused on the first path in this mediation and on nonclinical studies.

To our knowledge, there are no specific programs to date that target shame and guilt in children and adolescents. The current meta‐analysis supports the view that this would be a promising avenue for future studies. Hence, we advocate for broader programs with a dual focus on the parent‐child relationship and the child's emotional well‐being, such as the Positive Parenting Program (Triple P), which has been shown to be effective in promoting both healthy family interactions and child developmental outcomes (Sanders et al., 2014).

### Limitations of the primary studies

As this meta‐analysis draws on data derived predominantly from cross‐sectional studies (88.3%), more intensive and nonintensive longitudinal studies are needed. Moreover, only three of the included studies originated from a non‐Western cultural context, and within the U.S. samples, the majority of participants were White. Although shame and guilt are strongly influenced by culture (Wong & Tsai, 2007), we only found single‐study effects when testing differences between geographical regions and no significant effects of ethnicity within the U.S. samples. With regard to our sample, a cautious interpretation of these effects is appropriate, and we advocate cross‐cultural comparisons and sample diversification in future studies. Additionally, the measures used to assess shame and guilt in the included studies differed considerably in terms of their conceptualization and theoretical underpinnings, as did the measures used to assess the parent‐child relationship. Since the operationalization and the raters appeared to have an impact in the meta‐regressions, a critical review of the available assessment tools is needed in order to develop a more standardized procedure for assessing shame, guilt, and the parent‐child relationship across studies. Most measures applied were assessed in self‐report format, which may skew answers toward person‐specific theories of the assessed constructs as well as social acceptability. Lastly, shame and guilt overlap in terms of the situations that elicit them and the affective and physiological reactions to them (Tracy & Robins, 2004), which is why the respective other emotion is often statistically controlled for in quantitative studies (“shame‐free guilt” and “guilt‐free shame,” respectively; Tangney et al., 2009). We only used the uncontrolled Pearson's *r* in our analyses and note that the partial correlation coefficients provided by some of the original studies were usually larger or smaller.

### Limitations of this review

The inclusion of multiple conceptual indicators and measures for both the parent‐child relationship as well as shame and guilt might have created heterogeneity that might not be explainable by moderators and may limit the interpretations concerning specific effects. The aim of this meta‐analysis was to provide a comprehensive overview of the parent‐child relationship, but the broad categories of positive and dysfunctional characteristics may oversimplify this multifaceted topic. Despite conducting meta‐regressions to capture some nuances, our study, necessarily limited by the need to homogenize designs and variables across primary studies, may dilute the complex nature of these relationships.

Although we considered a range of theoretical as well as general methodological moderators, the few that reached significance varied across the meta‐analyses, with small effects overall. It is plausible to assume that we may have overlooked other relevant moderators (e.g., genetic factors, socio‐economic status, socializing experiences outside the parent‐child relationship), including those that were not explicitly reported in the included studies, as well as interactions between the moderators analyzed. The fact that the parent‐child relationship was assessed retrospectively in all adult samples, that is, the confounding of methodological aspects with sample characteristics, might further bias our findings. This is further evident for the confounding of measurement methods (e.g., behavioral coding) with the type of rater. In addition, due to the large number of studies identified in the initial search, we had to deviate from the preregistration in the screening and coding process. Although these changes were outlined in the protocol amendment before the final data analysis, this decision was made relatively late in the process of the meta‐analysis.

## CONCLUSION

This meta‐analysis synthesized the existing empirical evidence on the associations of the parent‐child relationship and nonpathological shame as well as adaptive and maladaptive guilt in children and adolescents. Positive parent‐child relationship characteristics were positively associated with adaptive guilt and negatively associated with shame. Conversely, indicators of a dysfunctional parent‐child relationship were found to be positively associated with child shame and maladaptive guilt. In sum, these findings emphasize the crucial role of the parent‐child relationship in children's social‐emotional development. Given that shame and guilt may mediate pathways from experiences in the parent‐child relationship to child mental health outcomes, we consider both the parent‐child relationship and the coping with feelings of shame and guilt as promising leverage points in the prevention and treatment of mental disorders in childhood and adolescence.

## AUTHOR CONTRIBUTIONS

R.L.E.: conceptualization, formal analysis, investigation, methodology, project administration, visualization, writing – original draft. M.S.: formal analysis, methodology, validation, visualization, writing – review & editing. A.J.J.: investigation. M.Z.: conceptualization, resources, supervision, writing – review & editing.

## CONFLICTS OF INTEREST

The authors declare no conflicts of interest.

## Supporting information


Data S1.

Data S2.

Data S3.

Data S4.

## Data Availability

This manuscript was drafted in compliance with the sociocultural policy of *Child Development*. Data, analytic code, and materials used and necessary to reproduce the analyses and replicate the findings in this article are publicly accessible and can be retrieved from OSF: https://osf.io/h9ge5/. This study was preregistered in a time‐stamped PRISMA‐P study protocol (Moher et al., 2015) on April 26, 2021 (https://osf.io/fnsxj), with protocol amendments on December 19, 2022 (https://osf.io/mdq43) and December 18, 2023 (https://osf.io/6cjxm). The PRISMA checklists for the protocol and this manuscript as well as all supplementary texts, figures, and tables mentioned throughout this manuscript are accessible in the electronic supplements (Data ESM1‐ESM3). The results of the meta‐analyses and *Q*‐tests for heterogeneity were submitted to and confirmed by StatCheck (Data ESM4).
