# Supplementary material for: The parent–child relationship and child shame and guilt: A meta‐analytic systematic review
Source: Child Dev. 2025 Jan 16;96(3):907–29. doi: 10.1111/cdev.14212 (PMC12023818; doi:10.1111/cdev.14212)
Supplement: Supplementary file 1 — Data S1. [file CDEV-96-907-s003.docx]

**The Parent-Child Relationship and Child Shame and Guilt: A Meta-Analytic Systematic Review**

**ESM 1**

**Text**

Date of first submission: February 01, 2024

## **CHE Model Rationale**

The CHE approach combines the advantages of the thus far applied hierarchical effects (HE) models, which allow for between- and within-study heterogeneity, and correlated effects (CE) models, which allow for correlated effect sizes, for meta-analysis. Pustejovsky and Tipton (2022) further advocate for a broader use of the CHE model as the correlated or hierarchical structure of data cannot be clearly determined in many cases, and meta-analytic datasets often contain a mixture of both.

## **Sample Retention**

Our first search yielded 14,718 results. After deduplication, we screened 8,003 records, of which we included 45 in the final meta-analysis. A backward search of the included studies of the first search yielded additional 62 records that we screened for eligibility, of which seven were included. An updated search of Web of Science in August 2023 yielded 960 new records, of which six were included.

Additionally, we wrote inquiries to seven authors, of which two replied and one (study ID 11) provided effect sizes. We recalculated the effect sizes of study IDs 2, 11, 50, and 57 from their effect sizes provided and newly calculated the effect sizes for study ID 23 from their open data on OSF. Five correlations of study IDs 4, 30, and 46 were reported as non-significant only (without specific values), which we coded as zero.

In total, 633 effect sizes from 65 samples (58 studies: 56 quantitative, one quasi-experimental, one qualitative) were included in our systematic review and meta-analysis.

## **Qualitative Results**

Only one qualitative study met our inclusion criteria and is briefly presented in narrative form, as preregistered. DeRobertis (2001) investigated the impact of psychological maltreatment during childhood in an empirical-phenomenological analysis of written abuse reports and following interviews with three adult participants. Throughout the reports, intense shame emerged as a recurring theme. The maternal figures were described as unreasonably critical, judgmental, and hurtful. The participants reported that the maltreatment induced feelings of being unwanted and unworthy of love and acceptance. Further, mothers’ verbal attacks were said to have instilled a sense of guilt that motivated dysfunctional self-prove. The inability to please their maternal figures lead participants to resignation and even self-destructive behavior.

**MMAT 2018**

Studies in which observations, conversation tasks, or transcribed quotations were analyzed using quantitative categories (deductively) and then treated statistically as numerical variables, were classified as quantitative descriptive instead of mixed methods studies according to the MMAT 2018 (Hong et al., 2018).

Study quality criteria according to the MMAT showed the greatest disparity for risk of non-response bias, followed by sampling approaches and representativeness of samples (see the ratings for MMAT4, MMAT1 and MMAT2 in Table S7 in the ESM 2). In 12.3% of all ratings (49.2% of samples), we were unable to assess with certainty whether a study met the criteria due to missing information in the publications. The respective criteria were rated as missing and thus not included in the respective meta-regression.

In one study that reported insufficiently on measurement (quality criteria MMAT3), the association between PPCR and adaptive guilt was weaker and the association of DPCR and adaptive guilt was significantly positive, although with a small effect. All other indicators of the MMAT showed no effect on the associations of interest. In the moderator analyses of study quality indicators, only the criterion of clear description of study measures (MMAT3) modulated effects, insofar as the study not fulfilling this criterion showed a weaker effect in the PPCR and guilt meta-analysis and a stronger effect in the DPCR and guilt meta-analysis. Since both effects were based on a single study, for which we further recoded the effect sizes, we consider this finding to be relatively negligible. Contrasts were not applied due to a lack of variation in the ratings of three quality criteria (MMATS1, MMATS2, and MMAT5), which were positively rated in all studies. The largest variance in ratings was found for the criteria of describing the target population (MMAT1) and the risk of missing data bias (MMAT4), although with no statistically significant differences in the outcomes.

**References**

*DeRobertis, E. M. (2000). *The long-term significance of having been psychologically maltreated by one’s maternal figure: An empirical- phenomenological investigation* [Unpublished doctoral dissertation]. Duquesne University.

Hong, Q. N., Gonzalez-Reyes, A., & Pluye, P. (2018). Improving the usefulness of a tool for appraising the quality of qualitative, quantitative and mixed methods studies, the mixed methods appraisal tool (MMAT). *Journal of Evaluation in Clinical Practice, 24*, 459–467. https://doi.org/10.1111/jep.12884

Pustejovsky, J. E., & Tipton, E. (2022). Meta-analysis with robust variance estimation: expanding the range of working models. *Prevention Science, 23*, 425–438. https://doi.org/10.1007/s11121-021-01246-3
