# Supplementary material for: The parent–child relationship and child shame and guilt: A meta‐analytic systematic review
Source: Child Dev. 2025 Jan 16;96(3):907–29. doi: 10.1111/cdev.14212 (PMC12023818; doi:10.1111/cdev.14212)
Supplement: Supplementary file 3 — Data S3. [file CDEV-96-907-s002.docx]

**The Parent-Child Relationship and Child Shame and Guilt: A Meta-Analytic Systematic Review**

**ESM 3**

**Figures**

Date of first submission: February 01, 2024

**Figure S1**

*Orchard Plot: Positive Parent-Child Relationship x Shame*


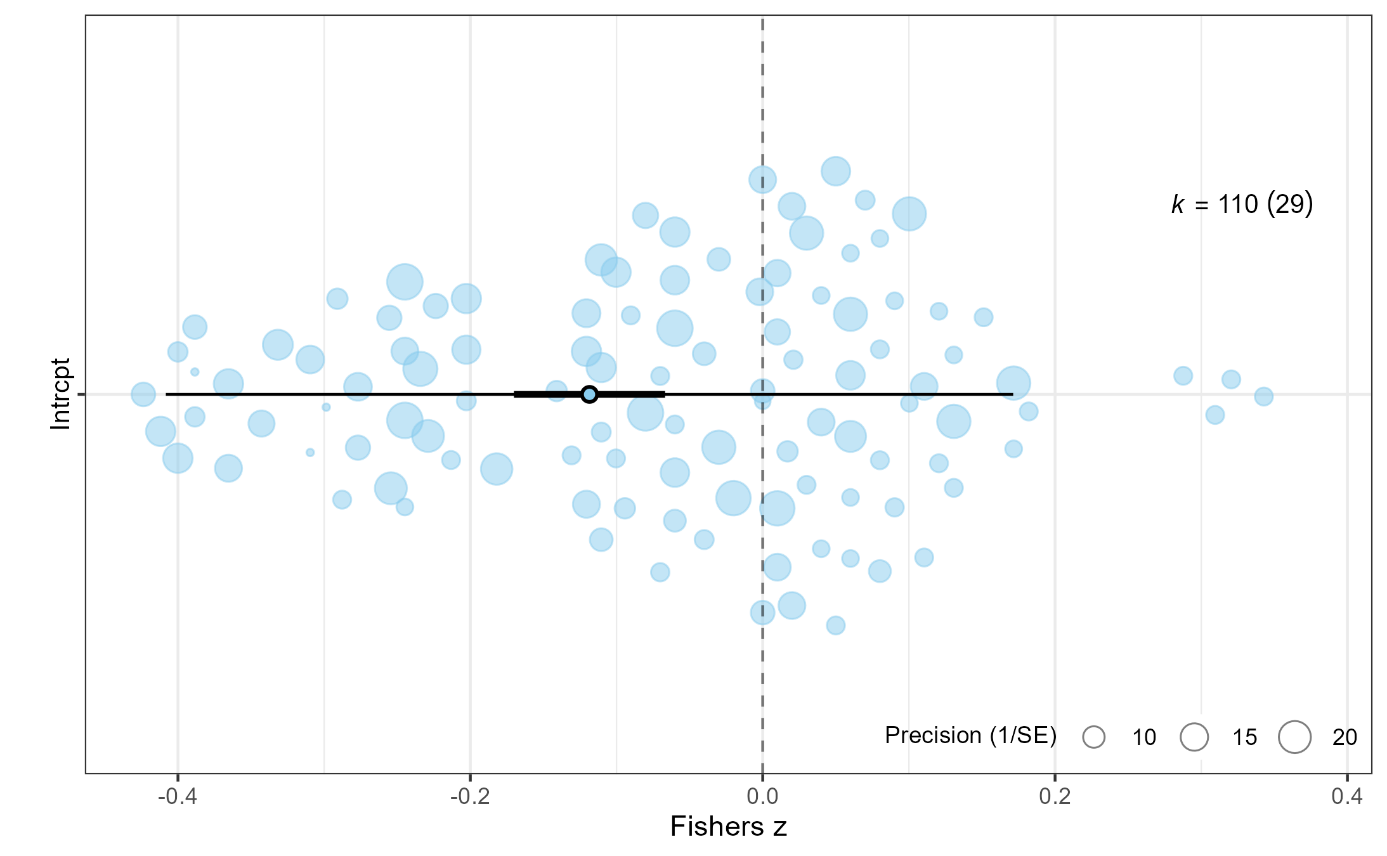


**Figure S2**

*Orchard Plot: Dysfunctional Parent-Child Relationship x Shame*


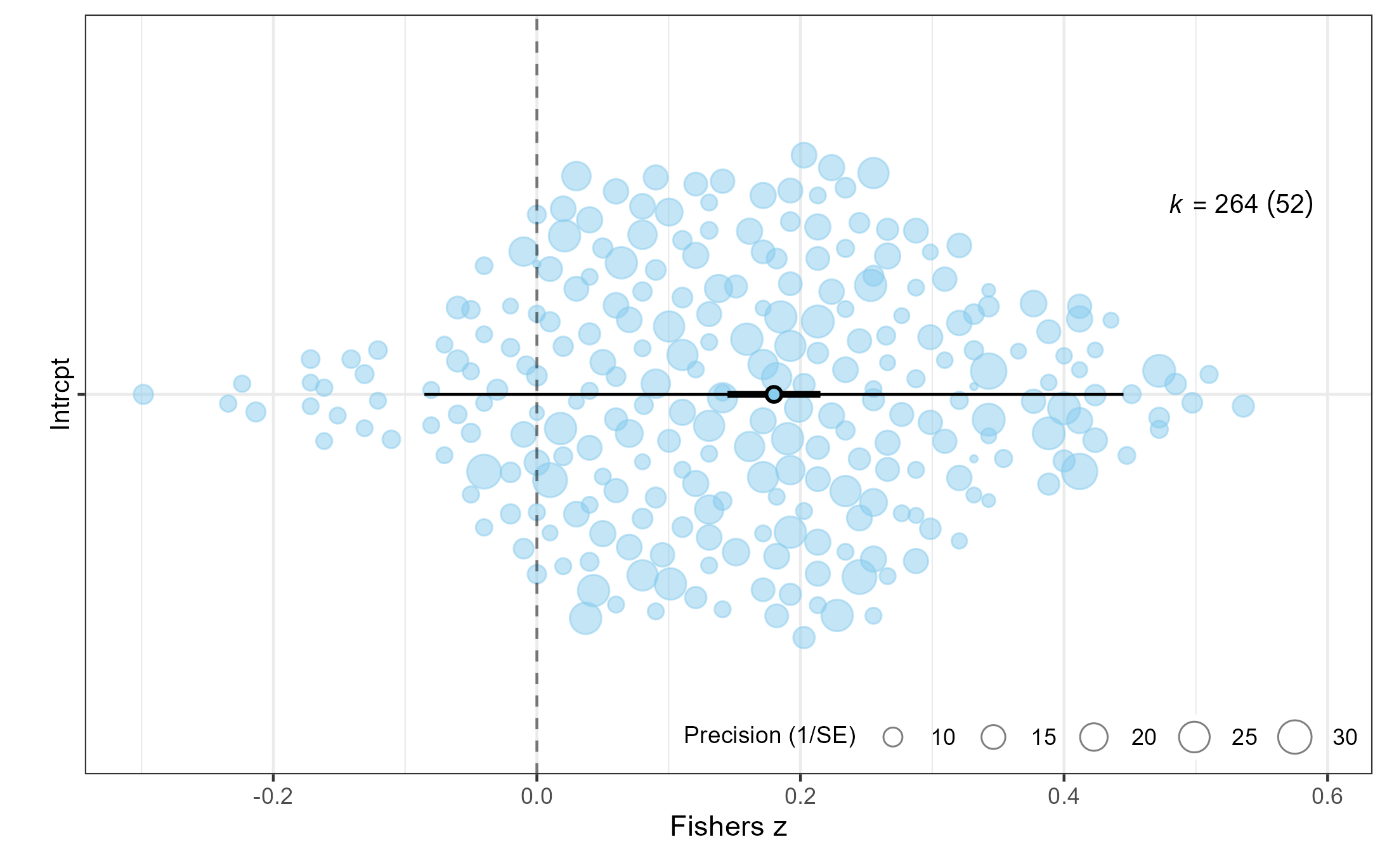


**Figure S3**

*Orchard Plot: Positive Parent-Child Relationship x Adaptive Guilt*


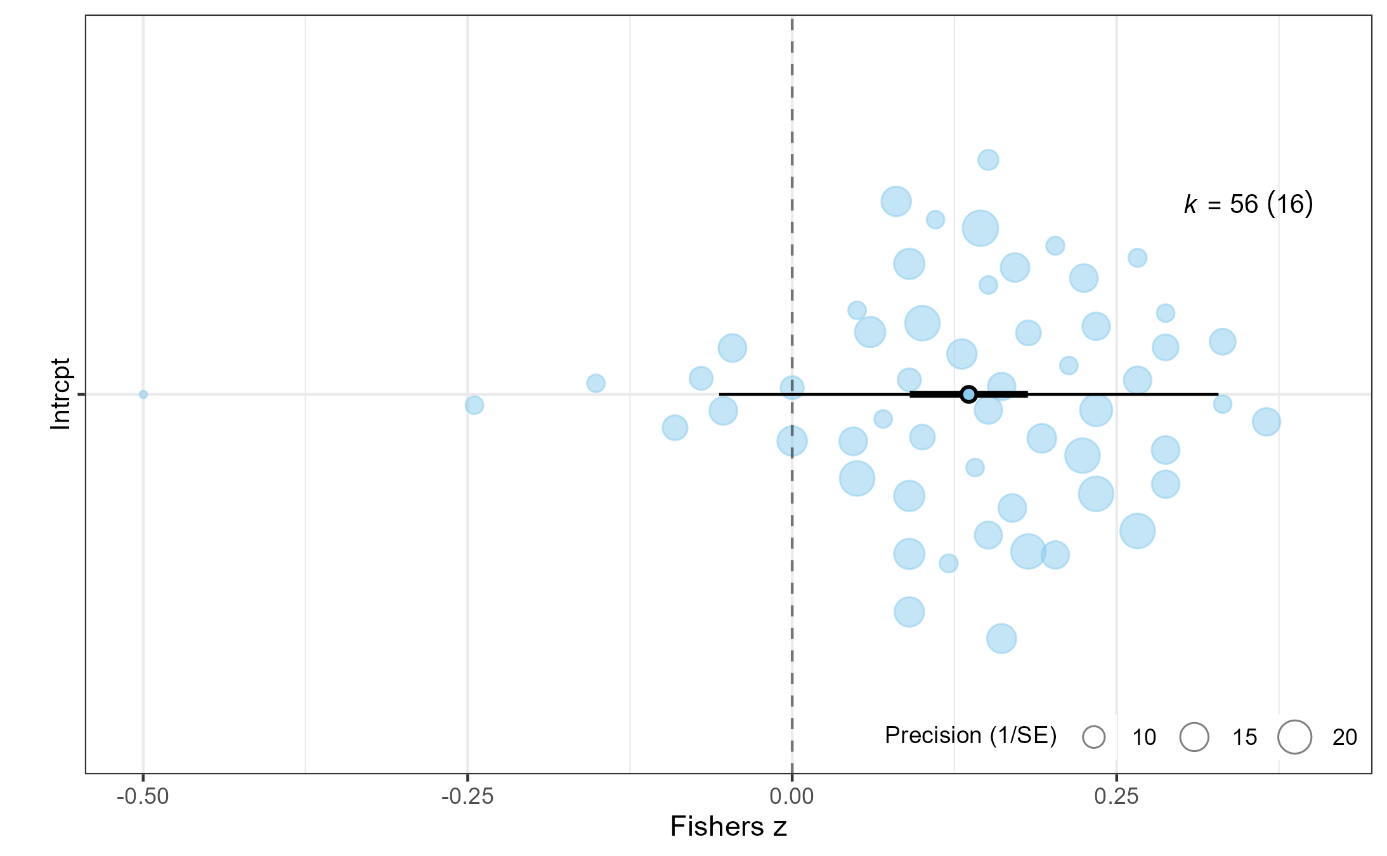


**Figure S4**

*Orchard Plot: Dysfunctional Parent-Child Relationship x Adaptive Guilt*


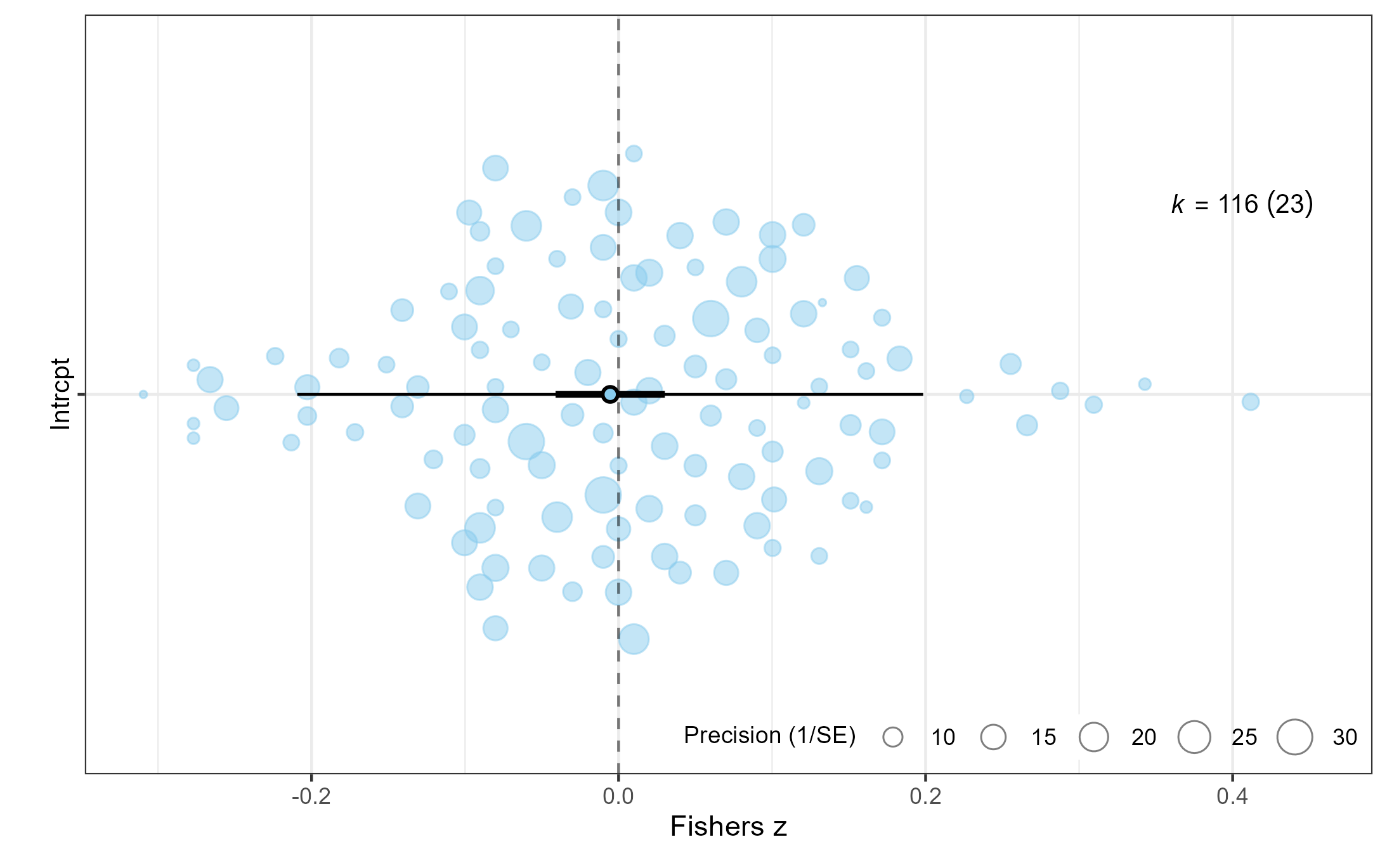


**Figure S5**

*Orchard Plot: Positive Parent-Child Relationship x Maladaptive Guilt*


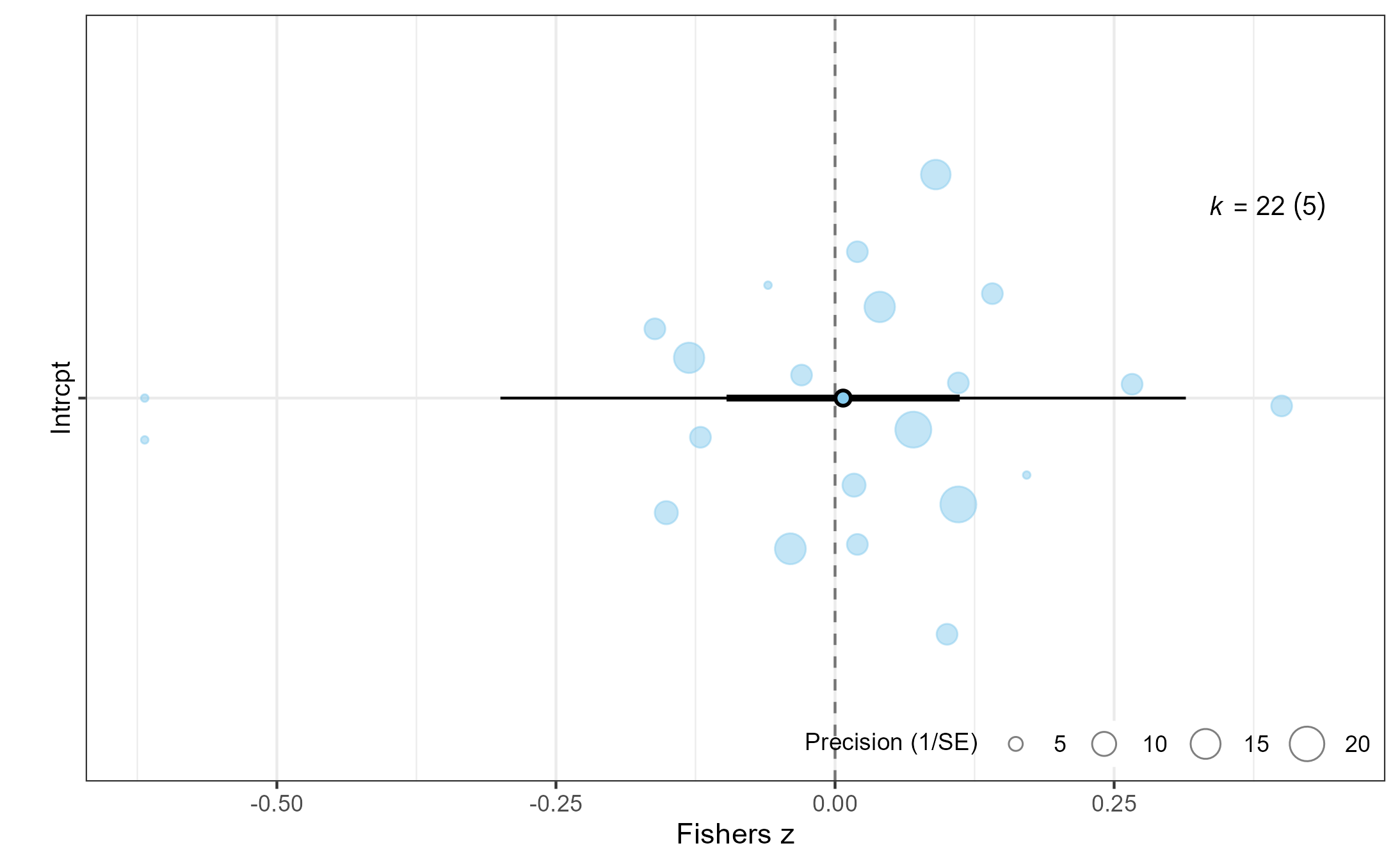


**Figure S6**

*Orchard Plot: Dysfunctional Parent-Child Relationship x Maladaptive Guilt*


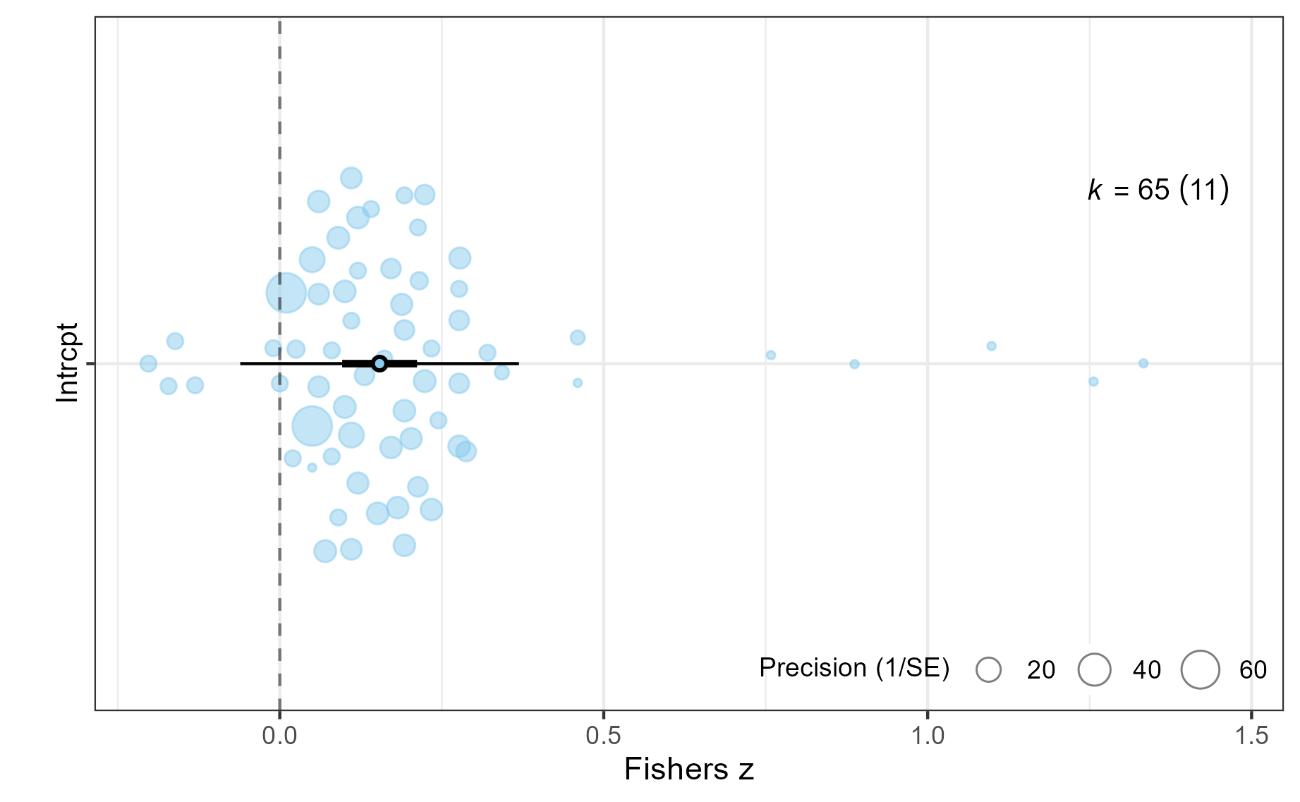


**Figure S7**

*Age Trend: Positive Parent-Child Relationship x Shame*


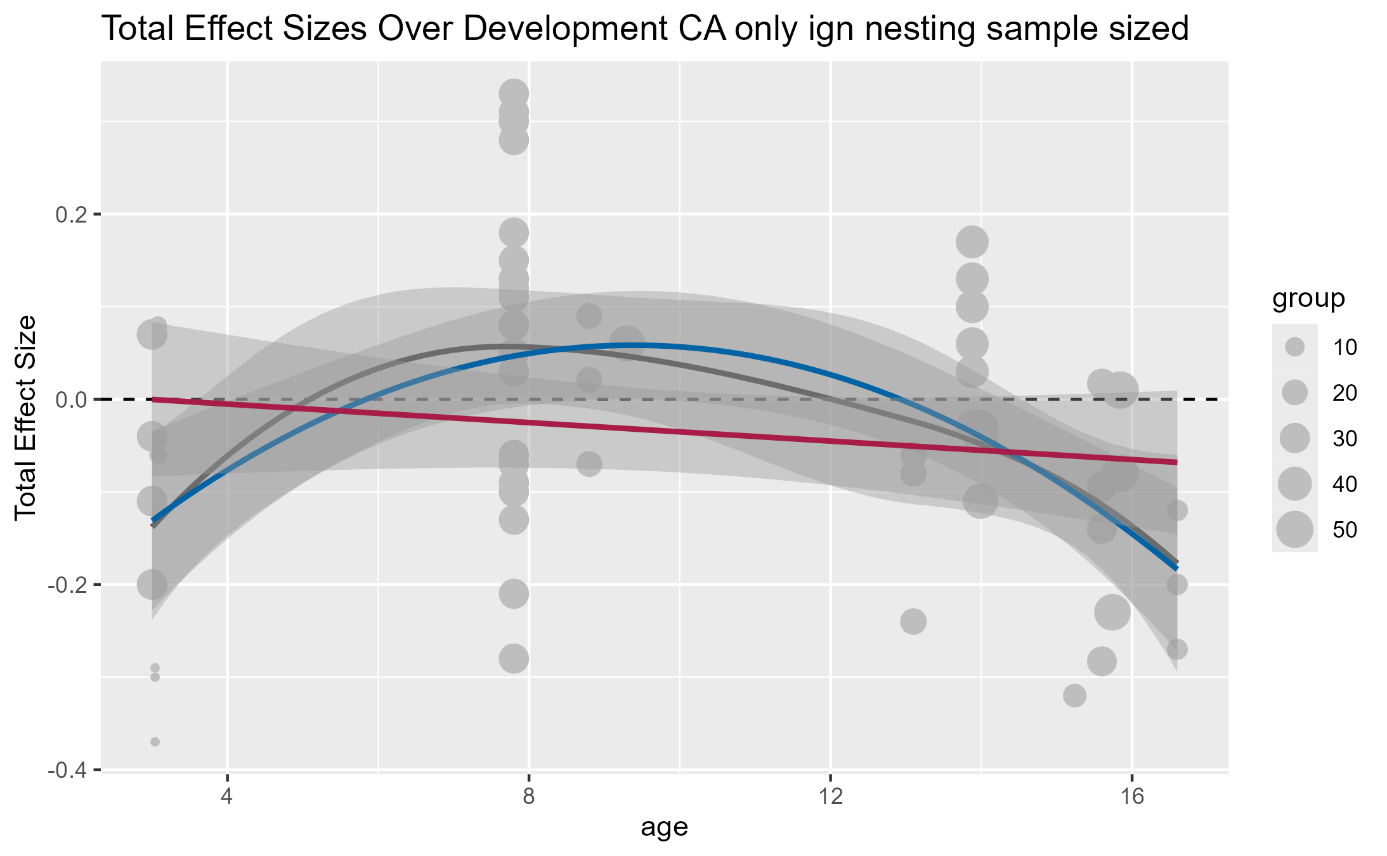


**Figure S8**

*Age Trend: Dysfunctional Parent-Child Relationship x Shame*


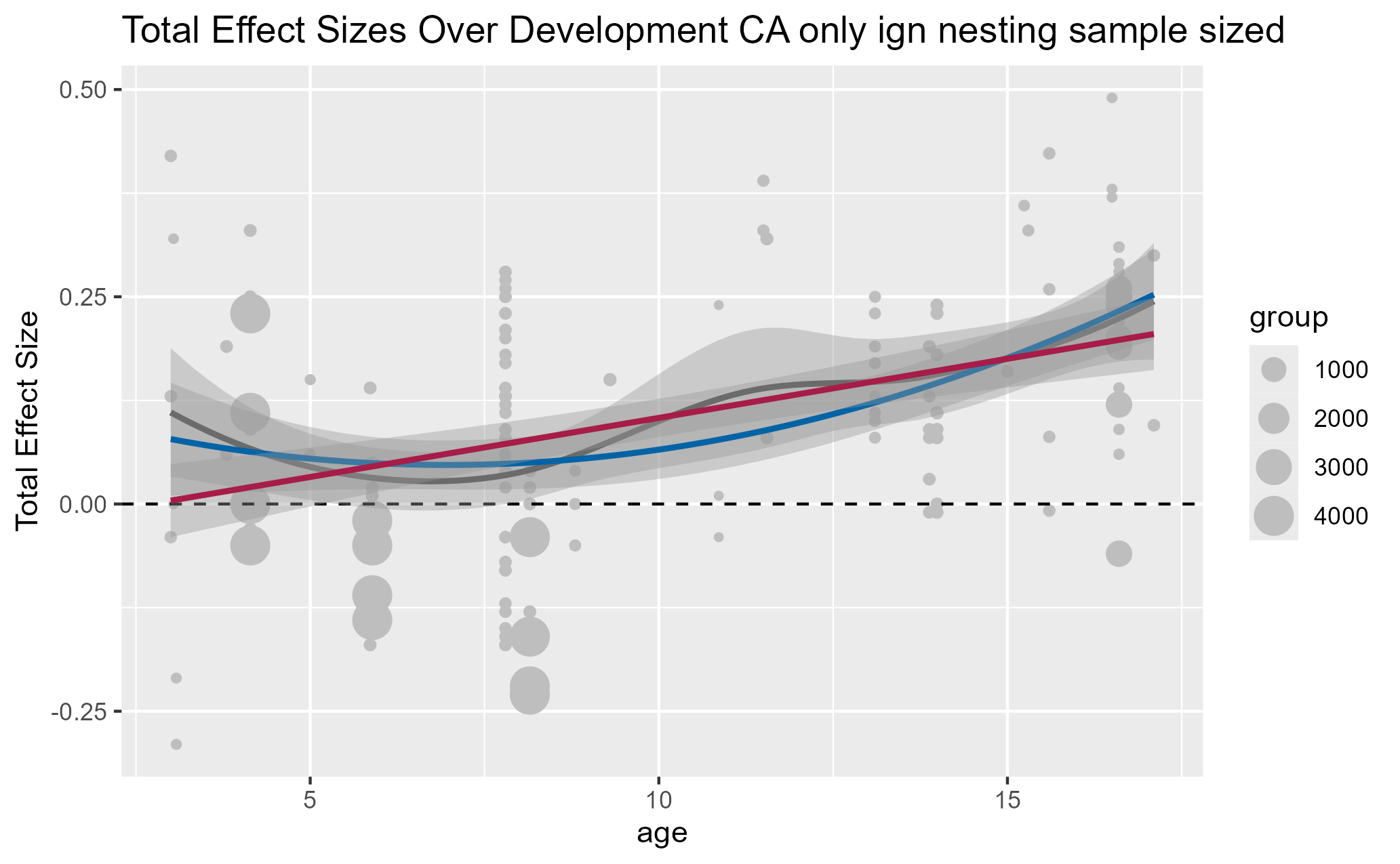


**Figure S9**

*Age Trend: Positive Parent-Child Relationship x Adaptive Guilt*


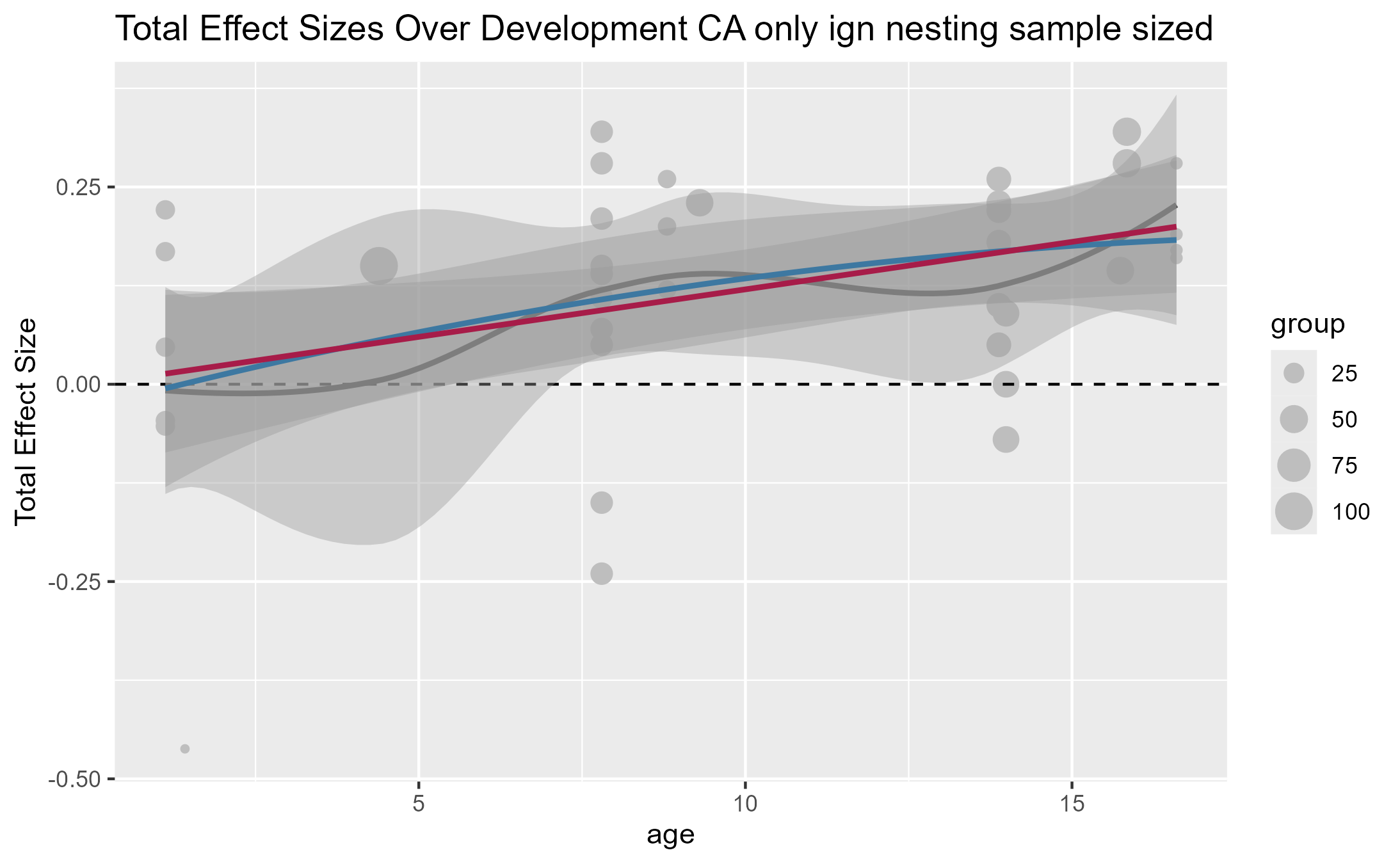


**Figure S10**

*Age Trend: Dysfunctional Parent-Child Relationship x Adaptive Guilt*


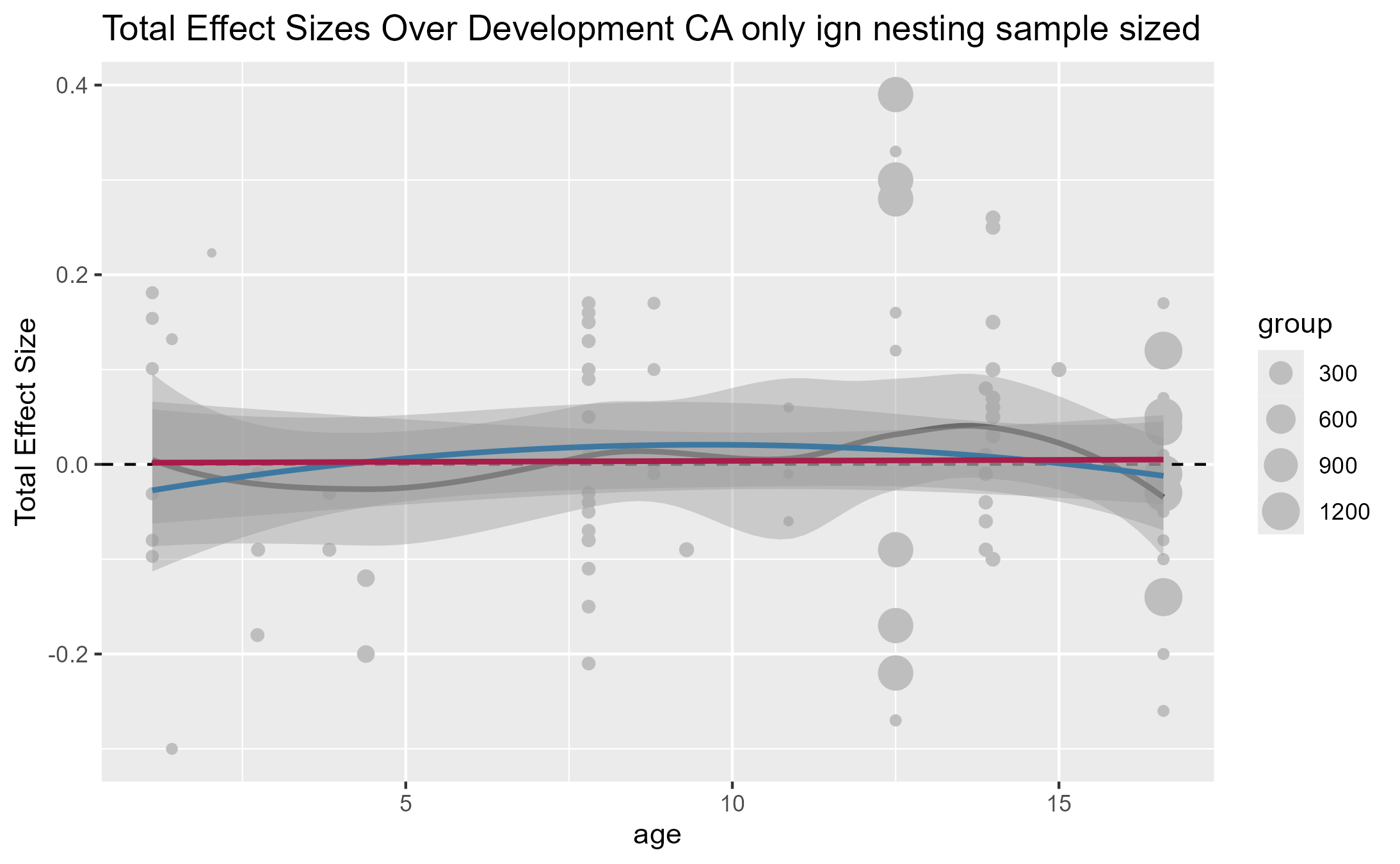


**Figure S11**


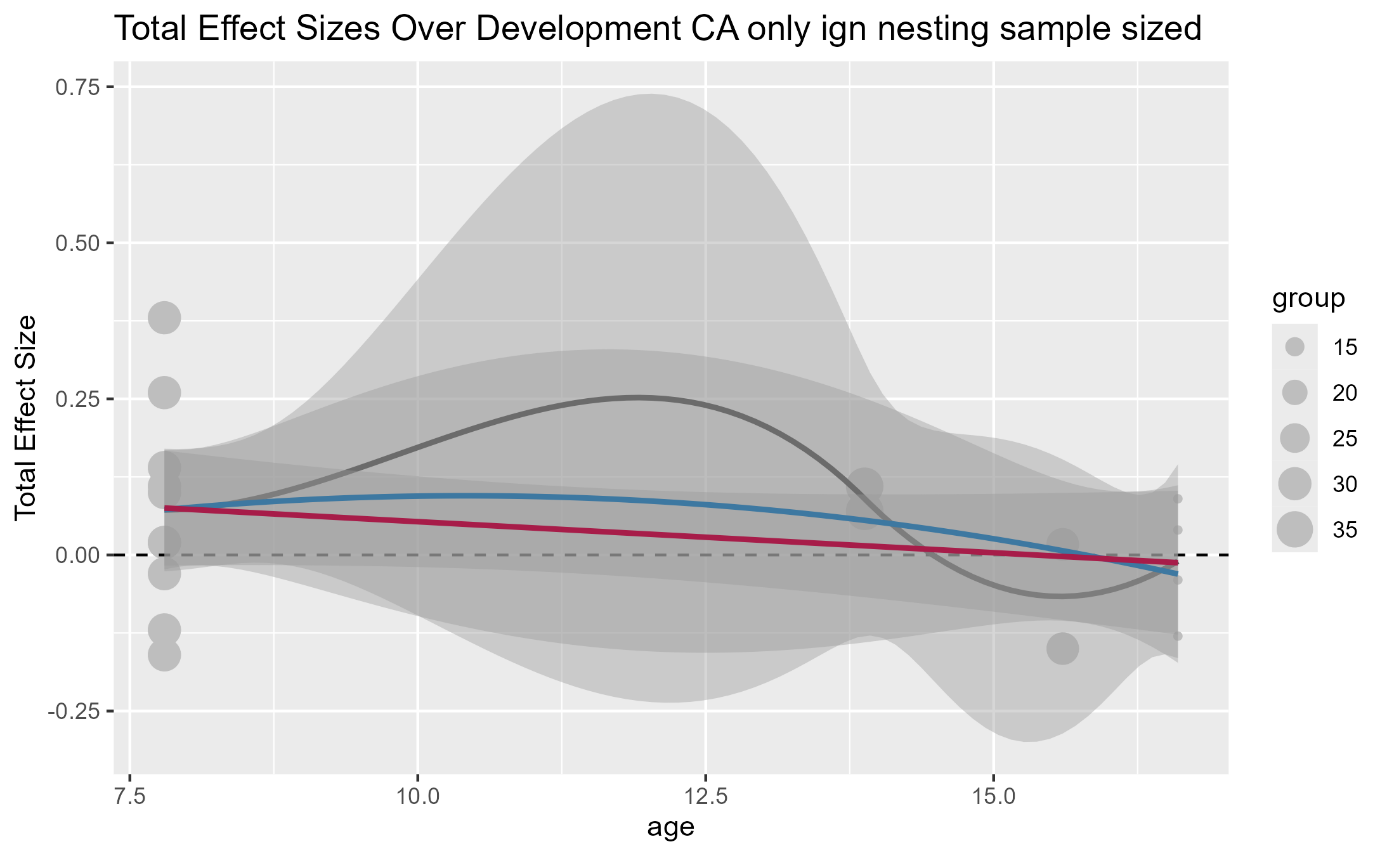
*Age Trend: Positive Parent-Child Relationship x Maladaptive Guilt*

**Figure S12**

*Age Trend: Dysfunctional Parent-Child Relationship x Maladaptive Guilt*


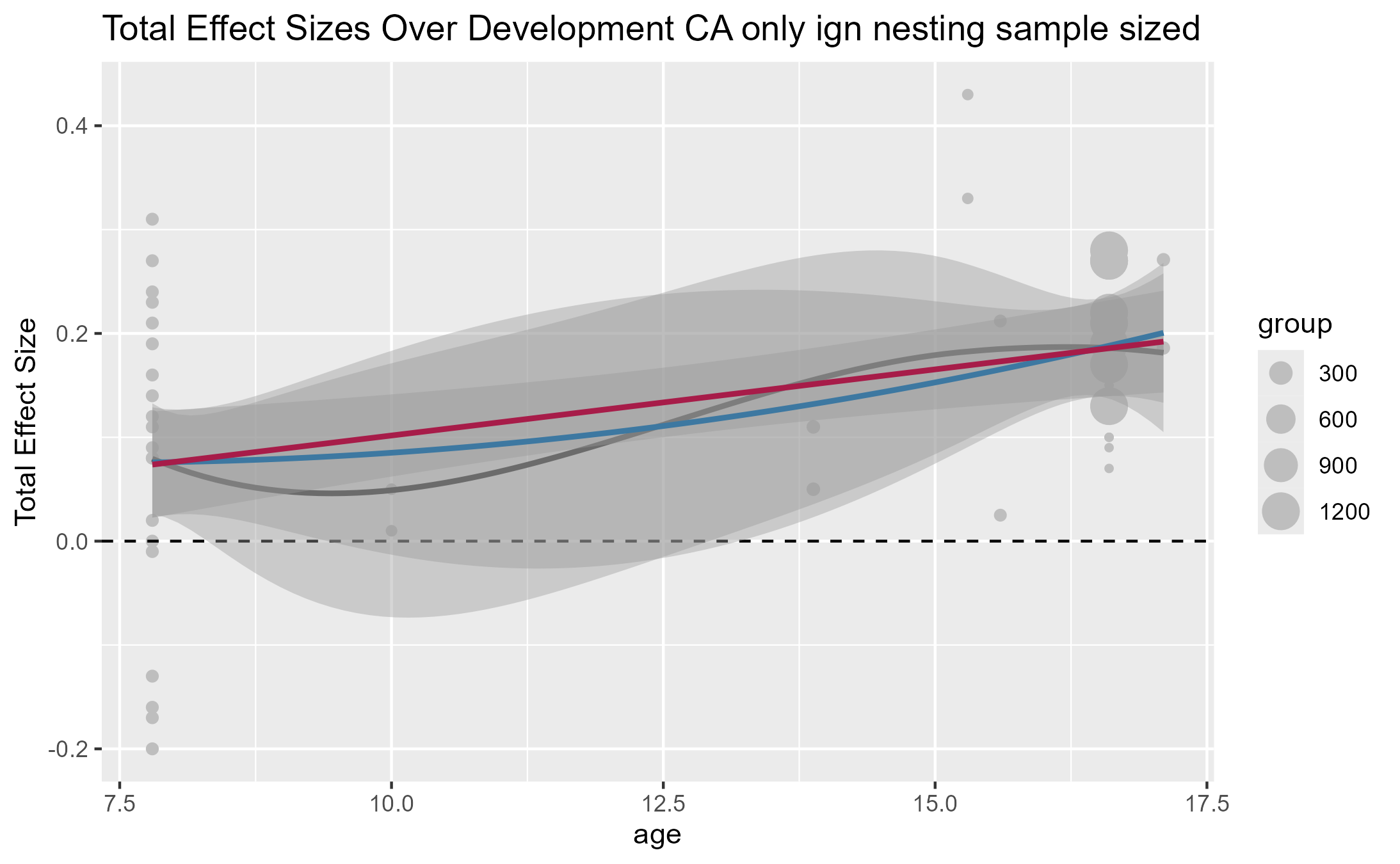


**Figure S13**

*Funnel Plot: Positive Parent-Child Relationship x Shame*


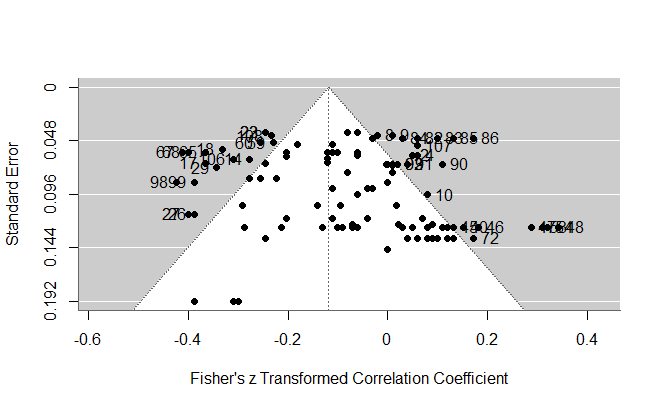


**Figure S14**

*Funnel Plot: Dysfunctional Parent-Child Relationship x Shame*


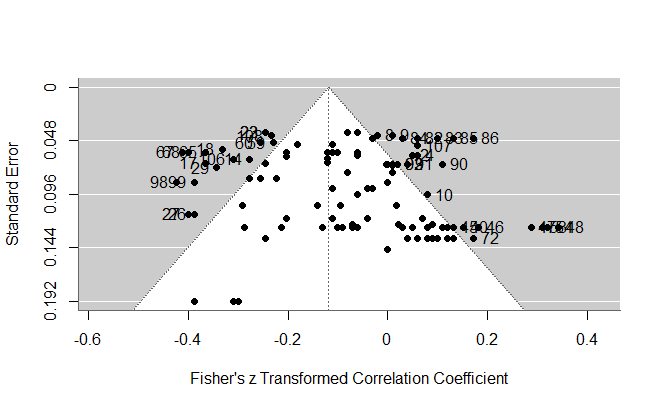


**Figure S15**

*Funnel Plot: Positive Parent-Child Relationship x Adaptive Guilt*


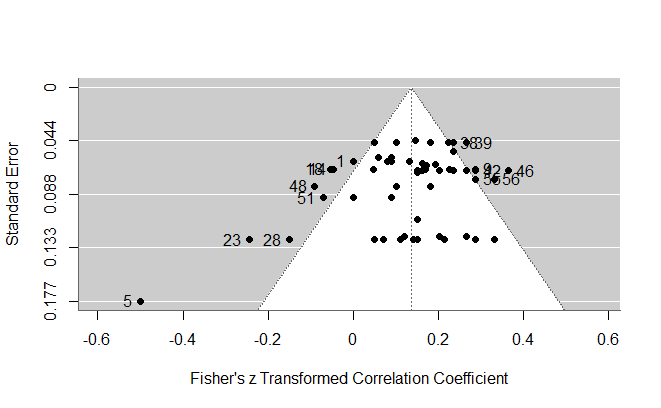


**Figure S16**

*Funnel Plot: Dysfunctional Parent-Child Relationship x Adaptive Guilt*


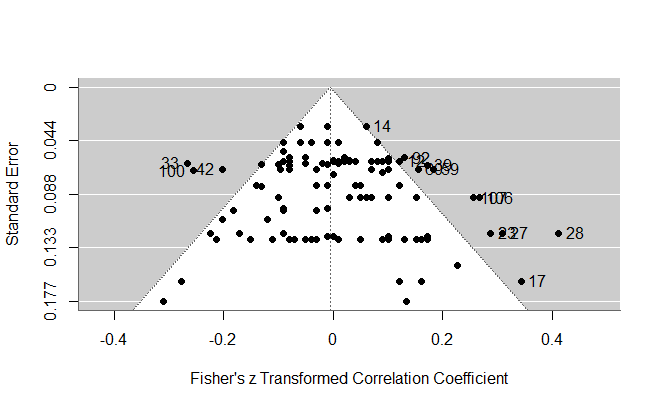


**Figure S17**

*Funnel Plot: Positive Parent-Child Relationship x Maladaptive Guilt*

**
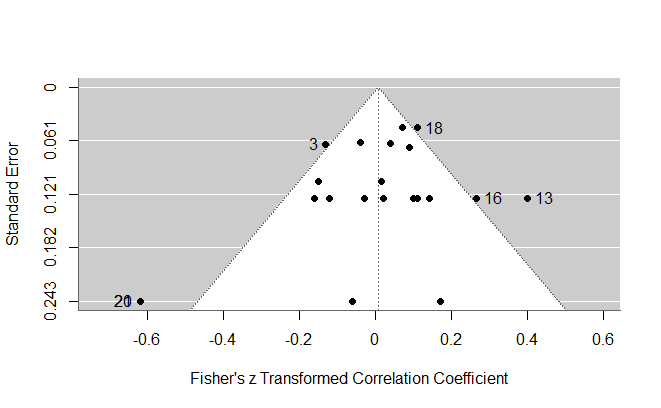
**

**Figure S18**

*Funnel Plot: Dysfunctional Parent-Child Relationship x Maladaptive Guilt*

*
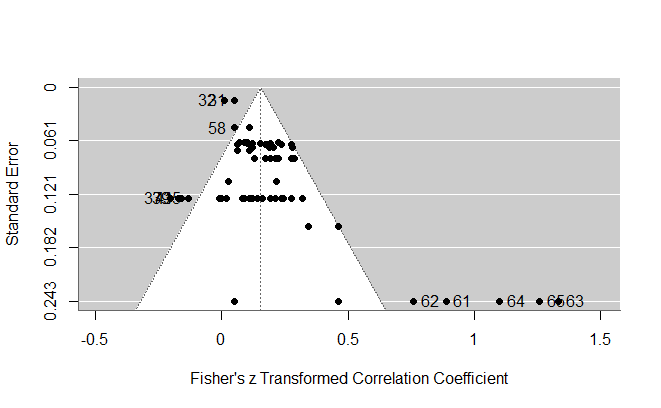
*

**Figure S19**

*Additional Funnel Plot: Dysfunctional Parent-Child Relationship x Maladaptive Guilt (excluding ID 54)*

*
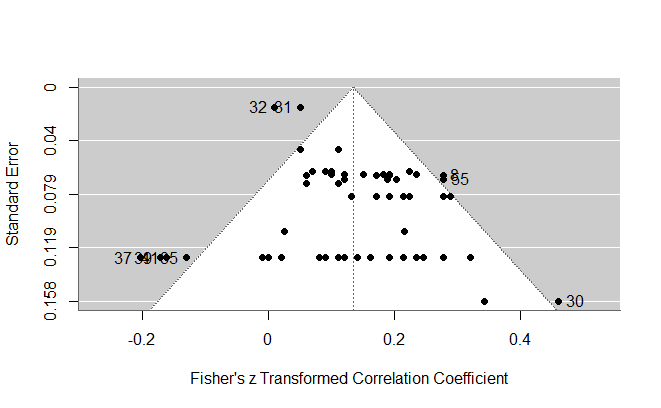
*

**Figure S20**

*Time Trend: Positive Parent-Child Relationship x Shame*

**
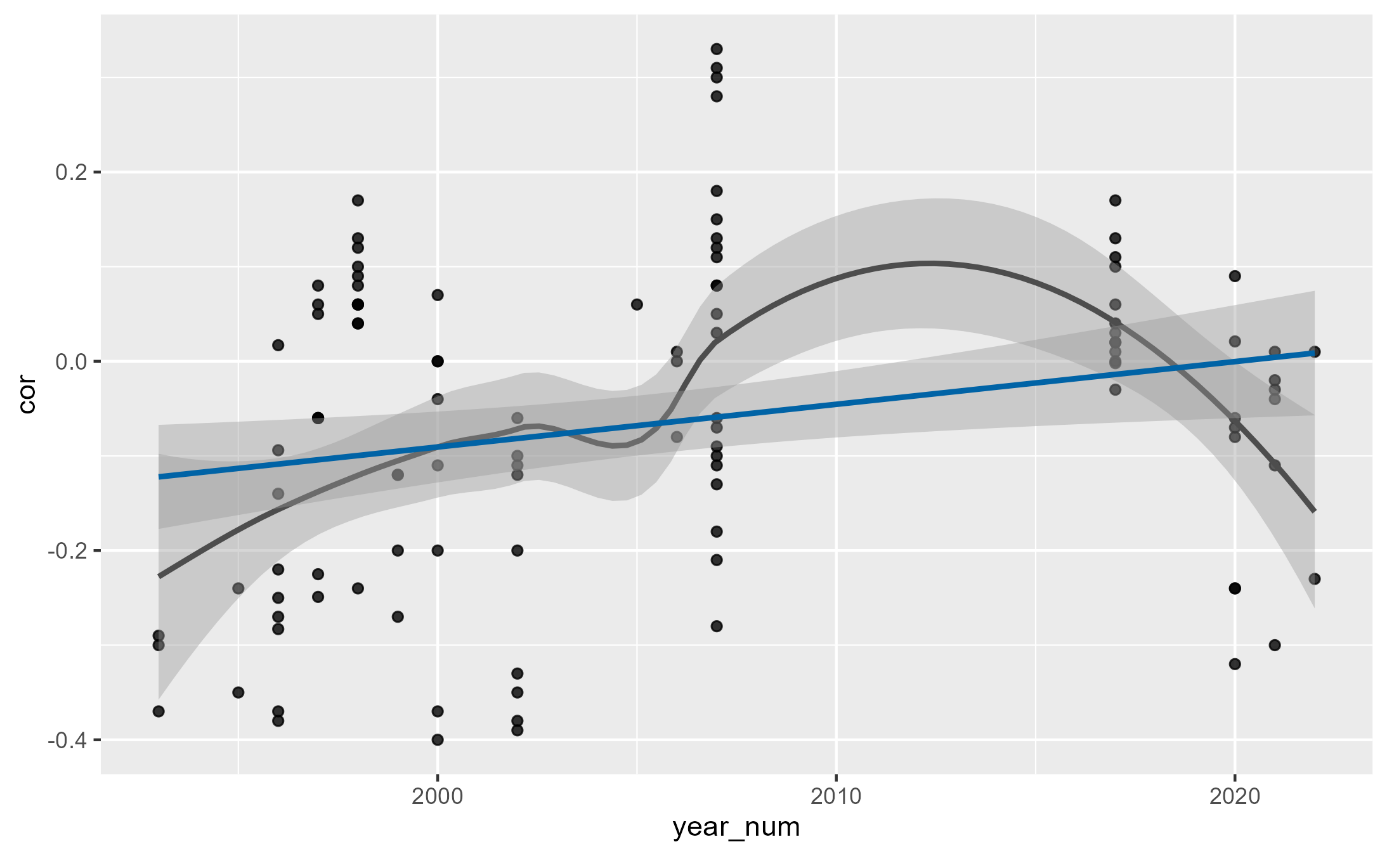
**

**Figure S21**

*Time Trend: Dysfunctional Parent-Child Relationship x Shame*


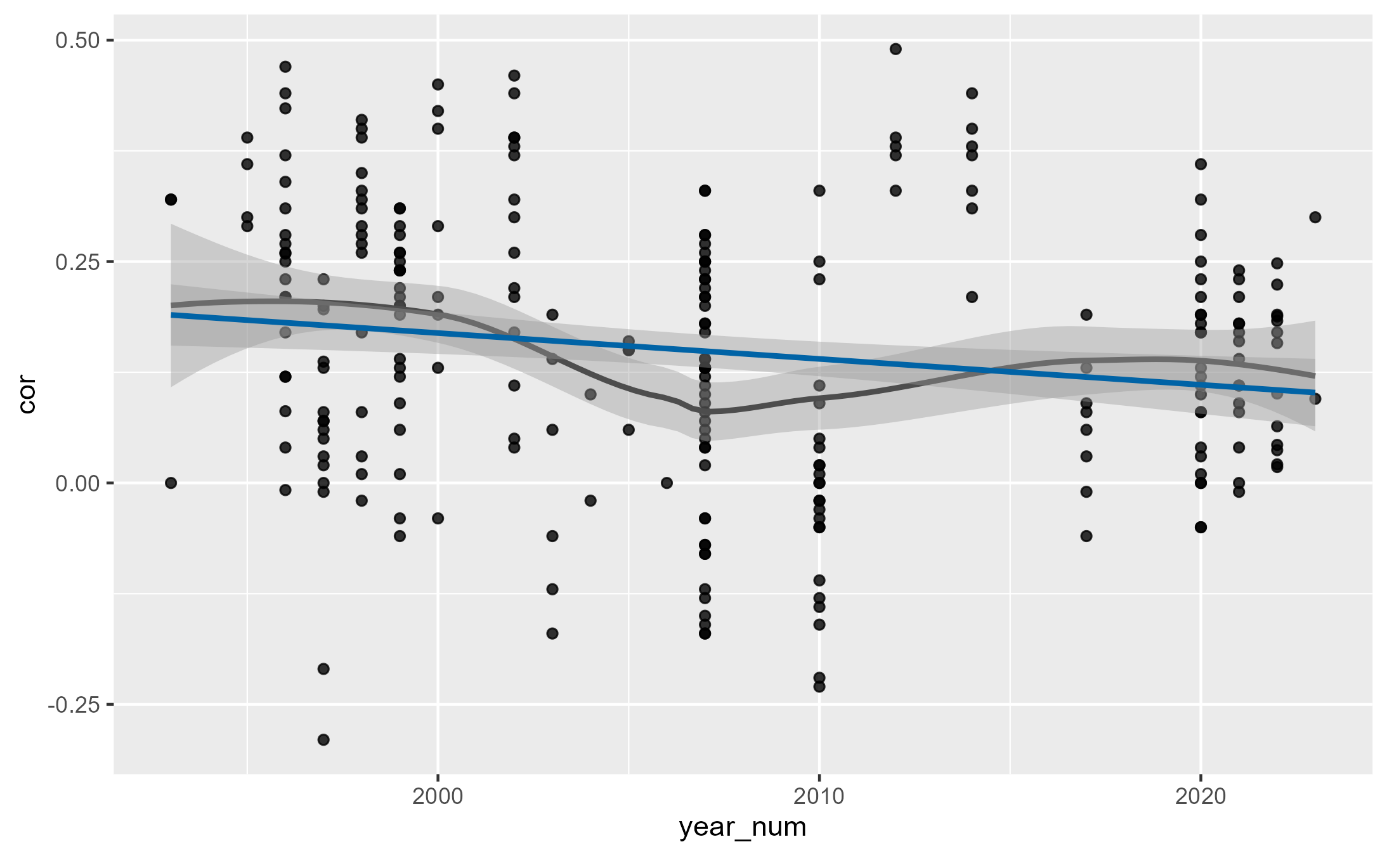


**Figure S22**

*Time Trend: Positive Parent-Child Relationship x Adaptive Guilt*


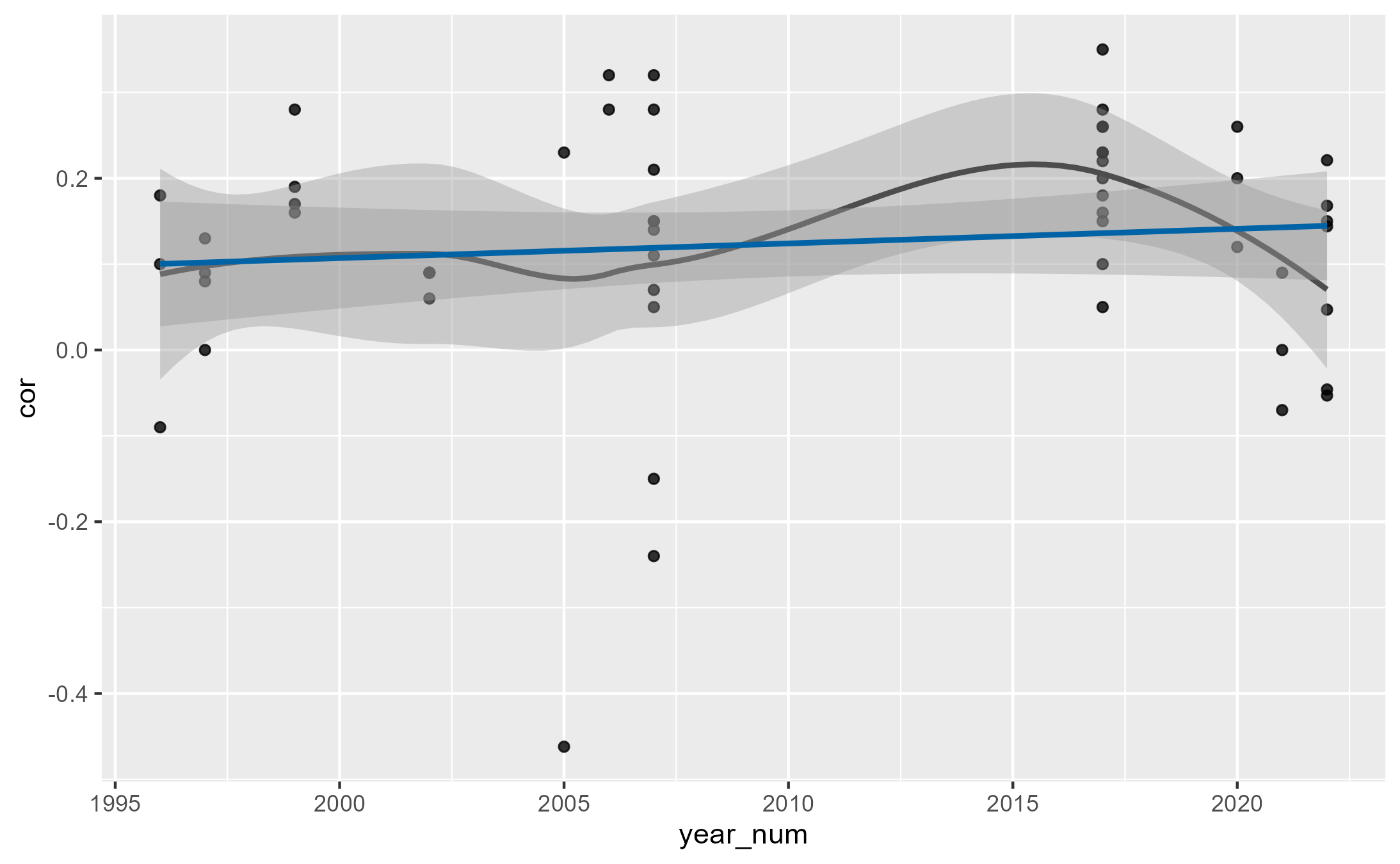


**Figure S23**

*Time Trend: Dysfunctional Parent-Child Relationship x Adaptive Guilt*


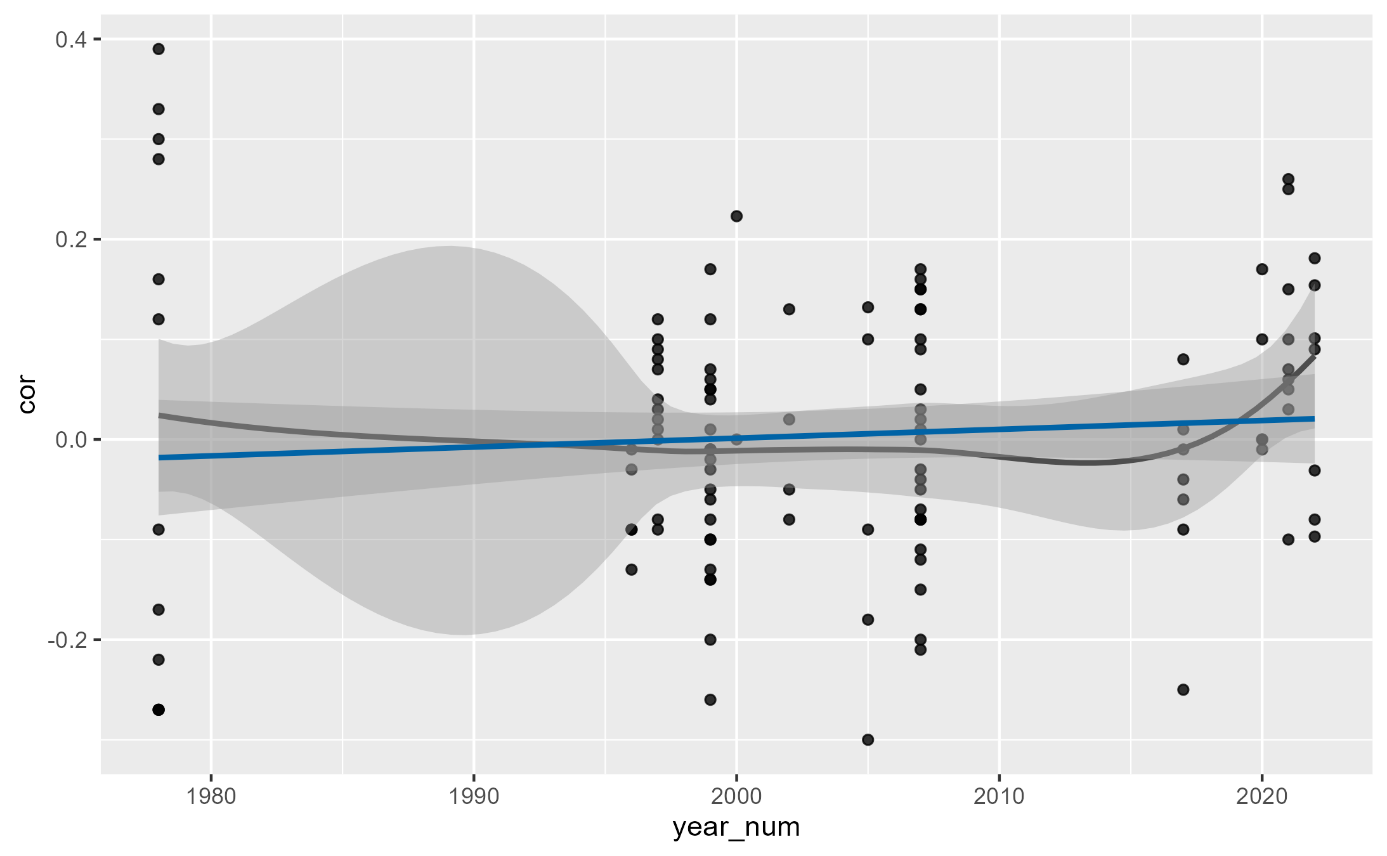


**Figure S24**

*Time Trend: Positive Parent-Child Relationship x Maladaptive Guilt*


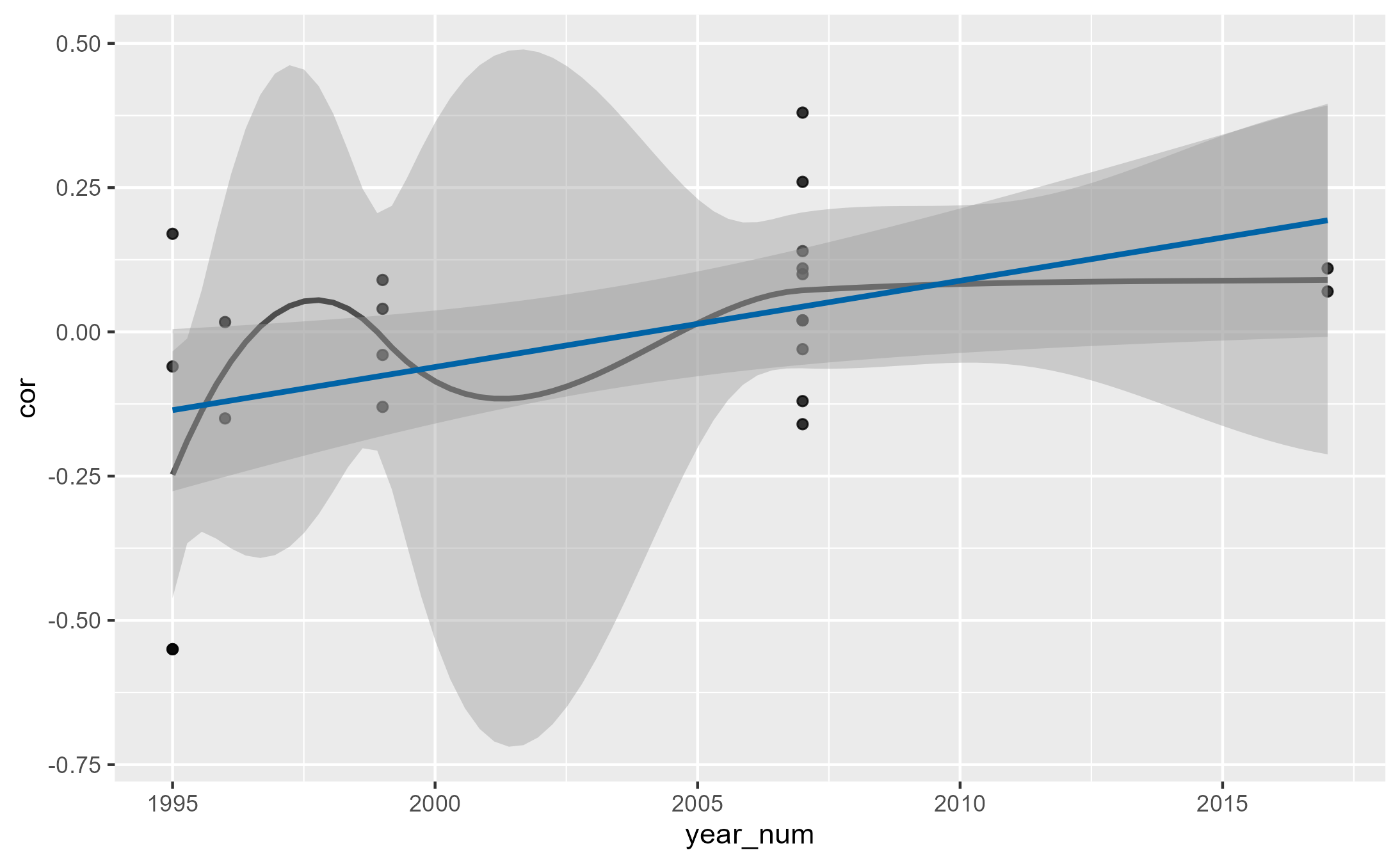


**Figure S25**

*Time Trend: Dysfunctional Parent-Child Relationship x Maladaptive Guilt*


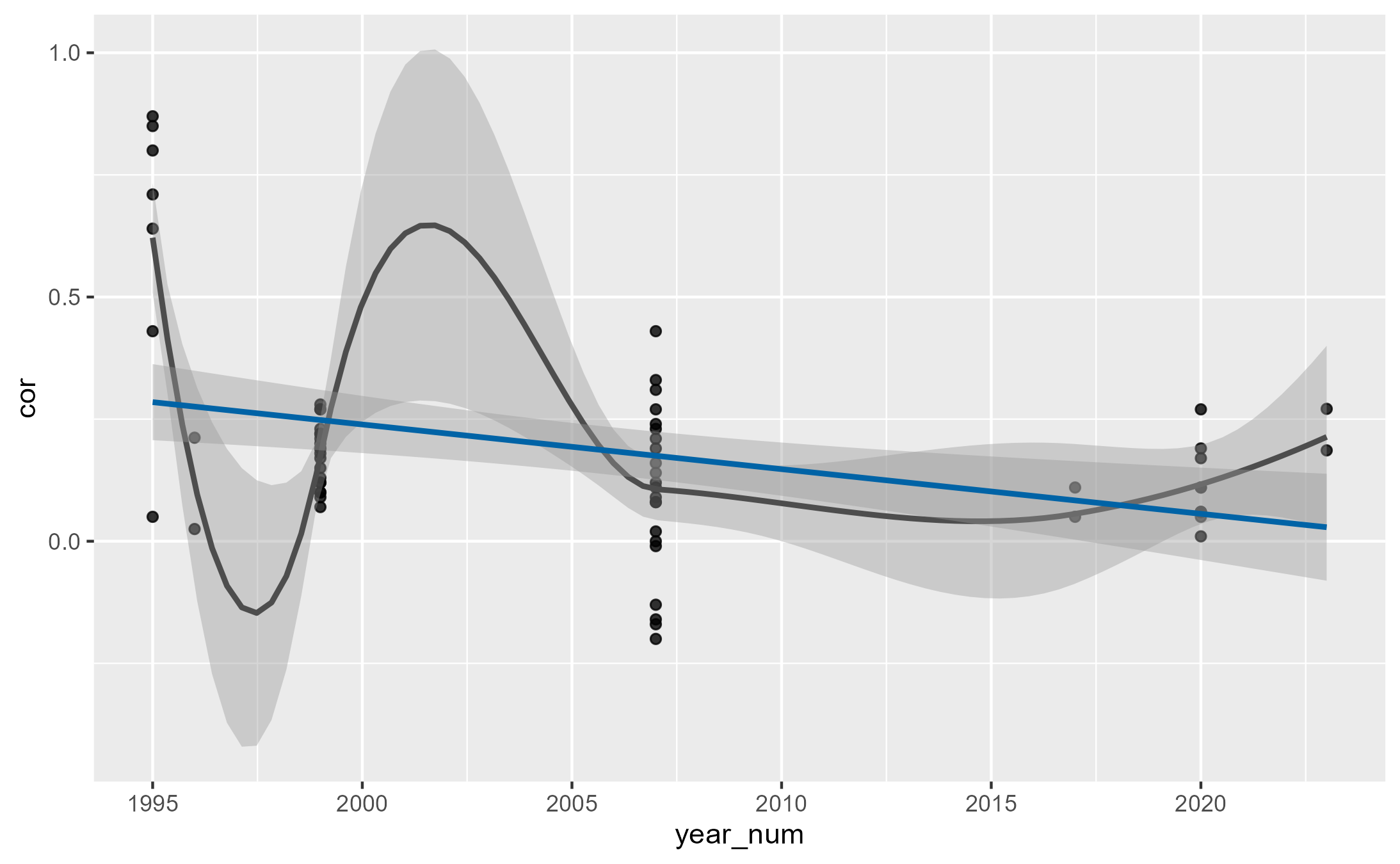


**References**

Nakagawa, S., Lagisz, M., O’Dea, R., Rutkowska, J., Yang, Y., Noble, D., & Senior, A. (2021). The orchard plot: Cultivating a forest plot for use in ecology, evolution and beyond [Preprint]. *Physical Sciences and Mathematics*. https://doi.org/10.32942/OSF.IO/EPQA7

Wickham, H. (2016). *ggplot2: Elegant graphics for data analysis*. Springer. https://ggplot2.tidyverse.org.
