# Supplementary material for: The parent–child relationship and child shame and guilt: A meta‐analytic systematic review
Source: Child Dev. 2025 Jan 16;96(3):907–29. doi: 10.1111/cdev.14212 (PMC12023818; doi:10.1111/cdev.14212)
Supplement: Supplementary file 4 — Data S4. [file CDEV-96-907-s004.docx]

**The Parent-Child Relationship and Child Shame and Guilt: A Meta-Analytic Systematic Review**

**ESM 4**

**StatCheck**

Date of first submission: February 01, 2024

**StatCheck**

The StatCheck input is based on the revised version of the manuscript “The Parent-Child Relationship and Child Shame and Guilt: A Meta-Analytic Systematic Review” as submitted to Child Development on October 9, 2024.

**Input**

***P-Values of Meta-Analytic Summary Effects***

The *p*-values of the meta-analytic summary effects are displayed in Table 4 in the manuscript, and *t*-statistic and degrees of freedom extracted from the statistical results running the respective code pcrsg metaanalysis metaregression.qmd. Results were transformed to APA style text results for this document.

PPCR x shame: *t*(26.21) = -4.49, *p* < .001

DPCR x shame: *t*(47.17) = 10.01, *p* < .001

PPCR x adaptive guilt: *t*(11.99) = 5.89, *p* < .001

DPCR x adaptive guilt: *t*(21.14) = -0.42, *p* = .681

PPCR x maladaptive guilt: *t*(3.15) = 0.20, *p* = .852

DPCR x maladaptive guilt: *t*(8.19) = 5.29, *p* = .001

***P-Values of Tests of Heterogeneity (Q-Test)***

The test statistics and *p*-values of the *Q*-test of heterogeneity are fully displayed in Table 4 in the manuscript and were transformed to APA style text results for this document.

PPCR x shame: *Q*(109) = 570.09, *p* < .001

DPCR x shame: *Q*(263) = 1429.42, *p* < .001

PPCR x adaptive guilt: *Q*(55) = 209.18, *p* < .001

DPCR x adaptive guilt: *Q*(115) = 484.30, *p* < .001

PPCR x maladaptive guilt: *Q*(21) = 88.24, *p* < .001

DPCR x maladaptive guilt: *Q*(64) = 236.52, *p* < .001

**Output**

The StatCheck output using the statistical information set out in the input section is displayed in Figure SC1.

**Figure SC1**


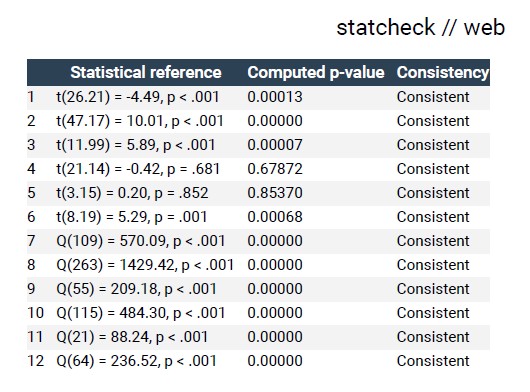
*StatCheck Results*

*Note.* Results as downloaded from statcheck.io using ESM 4, page 1, on July 16, 2024. *T* and *Q*-Statistics correspond to PPCR x shame (1, 7), DPCR x shame (2, 8), PPCR x adaptive guilt (3, 9), DPCR x adaptive guilt (4, 10), PPCR x maladaptive guilt (5, 11), and DPCR x adaptive guilt (6, 12). Inconsistencies on the second and third decimal are due to rounding errors in the analysis.
